# Supplementary figures and images for: HIF-1α induces glycolytic reprograming in tissue-resident alveolar macrophages to promote cell survival during acute lung injury
Source: eLife. 2022 Jul 13;11:e77457. doi: 10.7554/eLife.77457 (PMC9323005; doi:10.7554/eLife.77457)

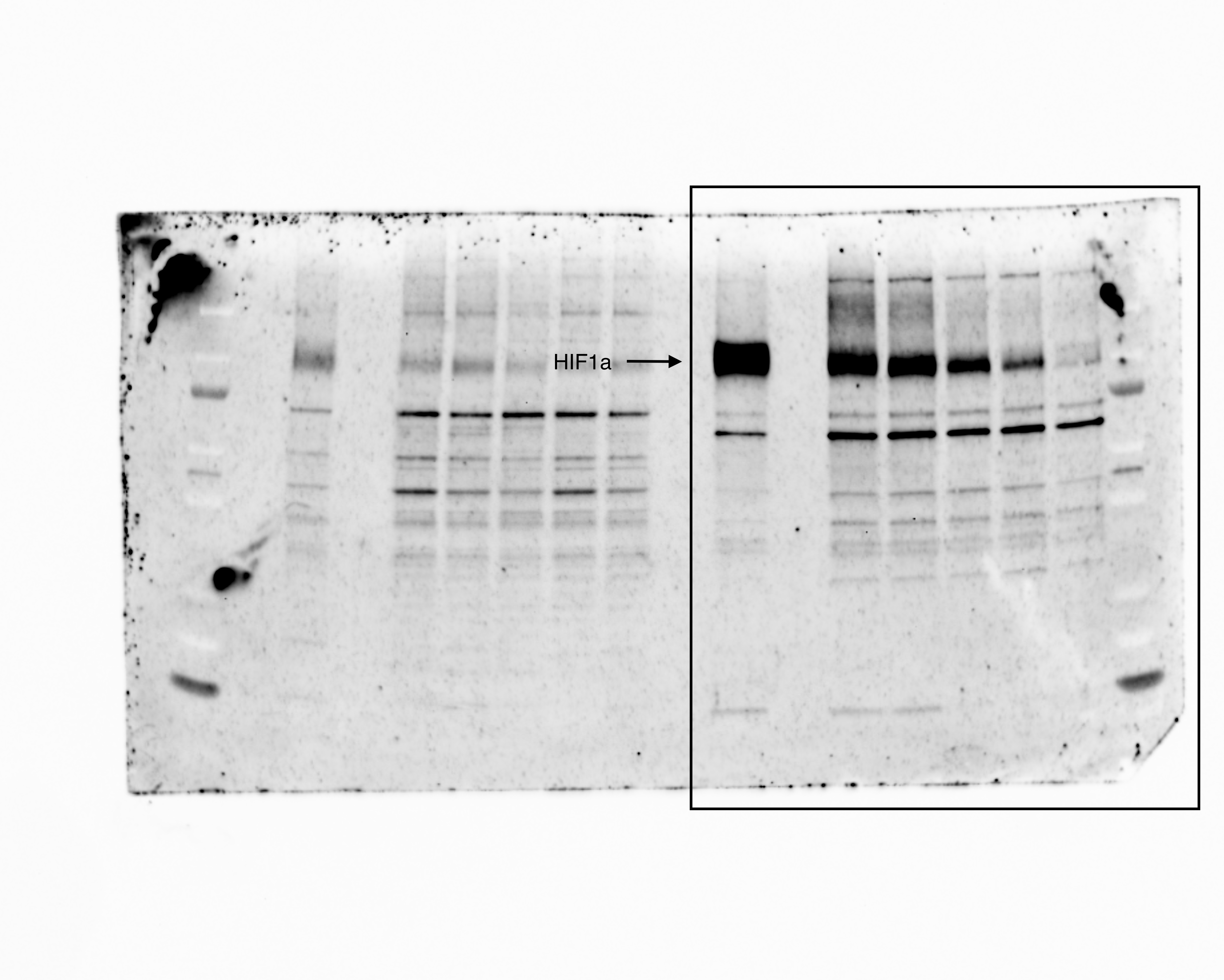

Supplement: Figure 1—source data 1. — Uncropped Western blot images of HIF-1α protein expression in TR-AMs under different concentrations of oxygen. [file elife-77457-fig1-data1.zip › Figure 1-source data 1 (Figure 1C)/Figure 1C-HIF1a.tif]

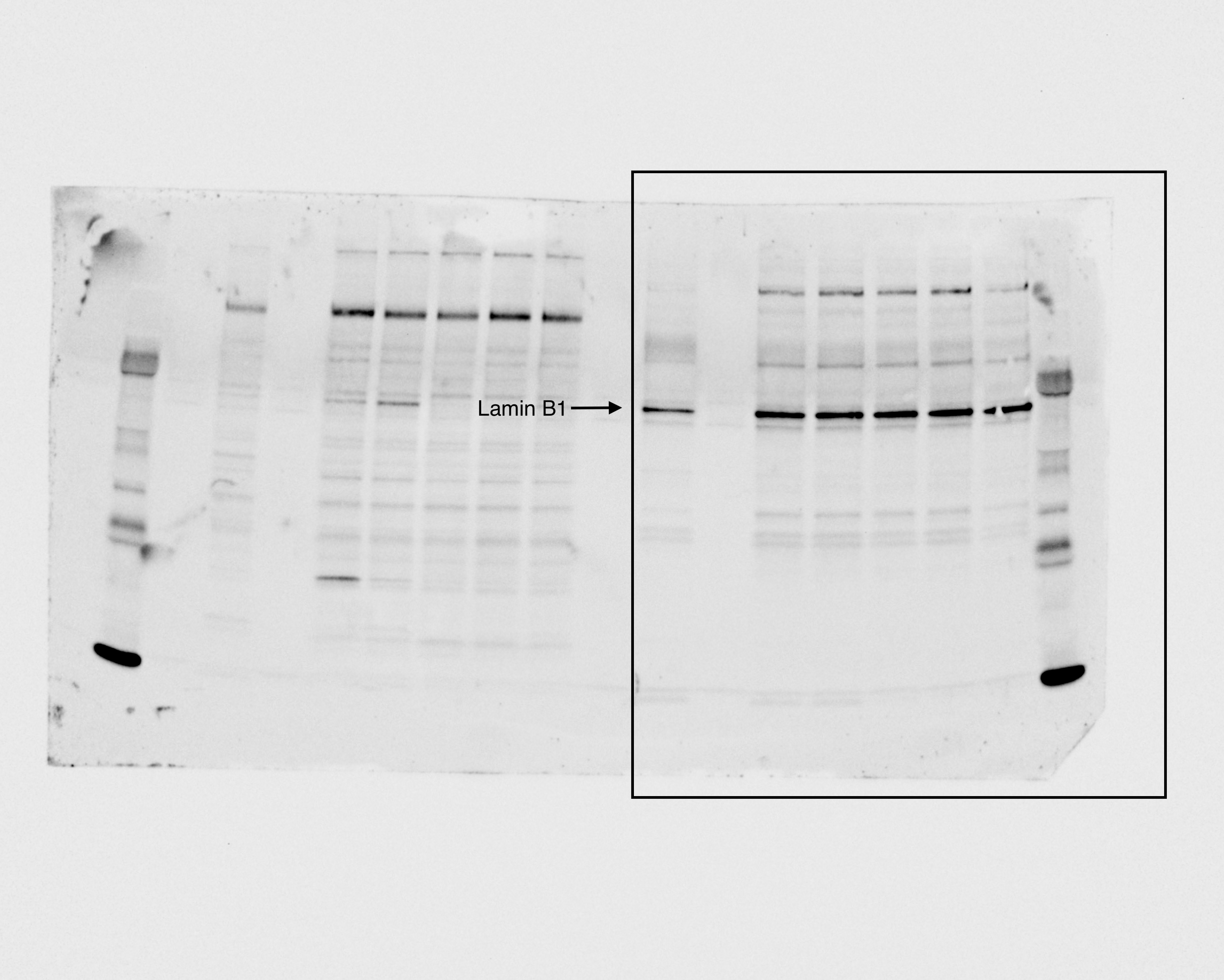

Supplement: Figure 1—source data 1. — Uncropped Western blot images of HIF-1α protein expression in TR-AMs under different concentrations of oxygen. [file elife-77457-fig1-data1.zip › Figure 1-source data 1 (Figure 1C)/Figure 1C-LaminB1.tif]

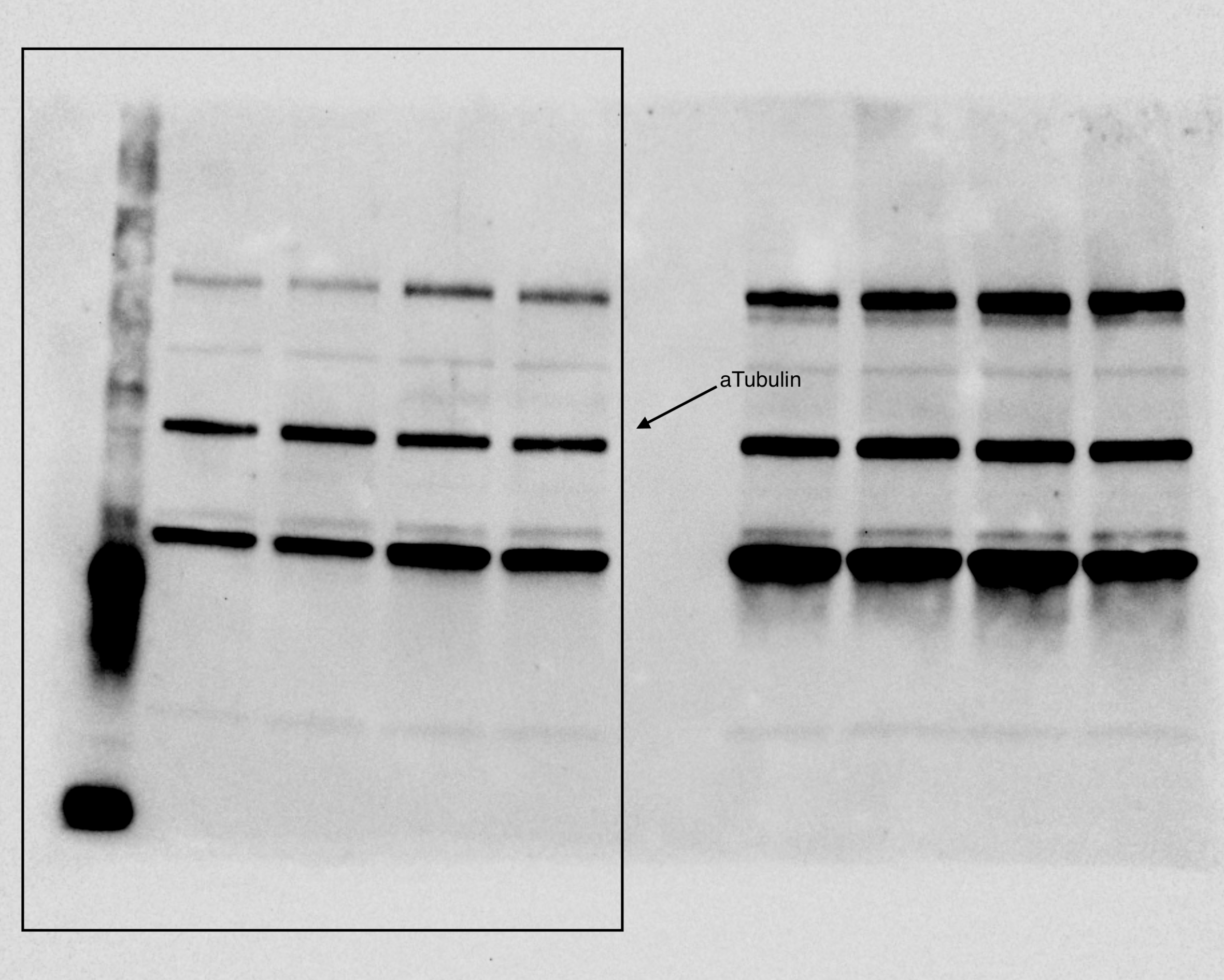

Supplement: Figure 1—source data 2. — Uncropped Western blot images of HK2, LDHA, and α-tubulin in TR-AMs treated with echinomycin under normoxia or hypoxia. [file elife-77457-fig1-data2.zip › Figure 1-source data 2 (Figure 1E)/Figure 1E-aTubulin.tif]

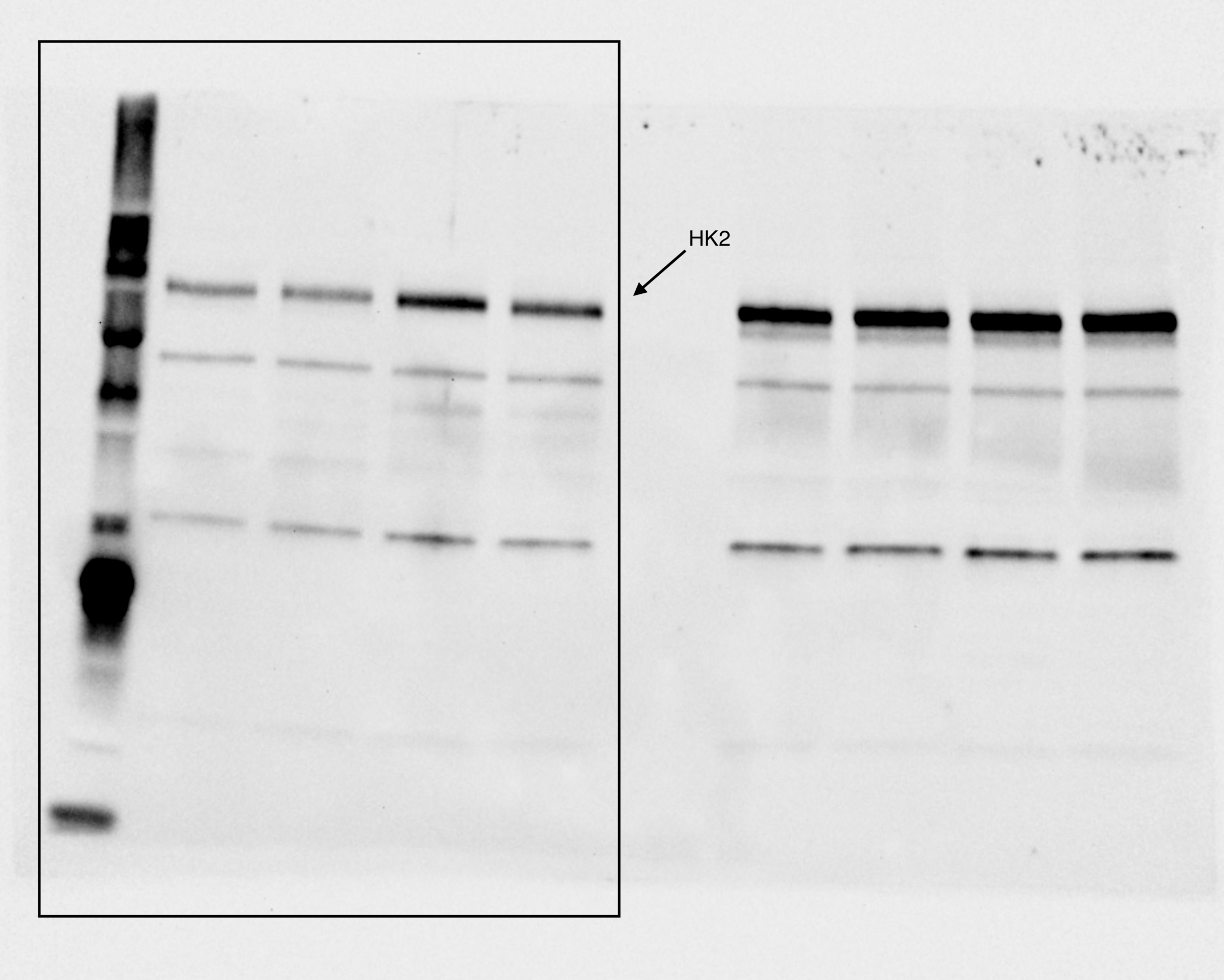

Supplement: Figure 1—source data 2. — Uncropped Western blot images of HK2, LDHA, and α-tubulin in TR-AMs treated with echinomycin under normoxia or hypoxia. [file elife-77457-fig1-data2.zip › Figure 1-source data 2 (Figure 1E)/Figure 1E-HK2.tif]

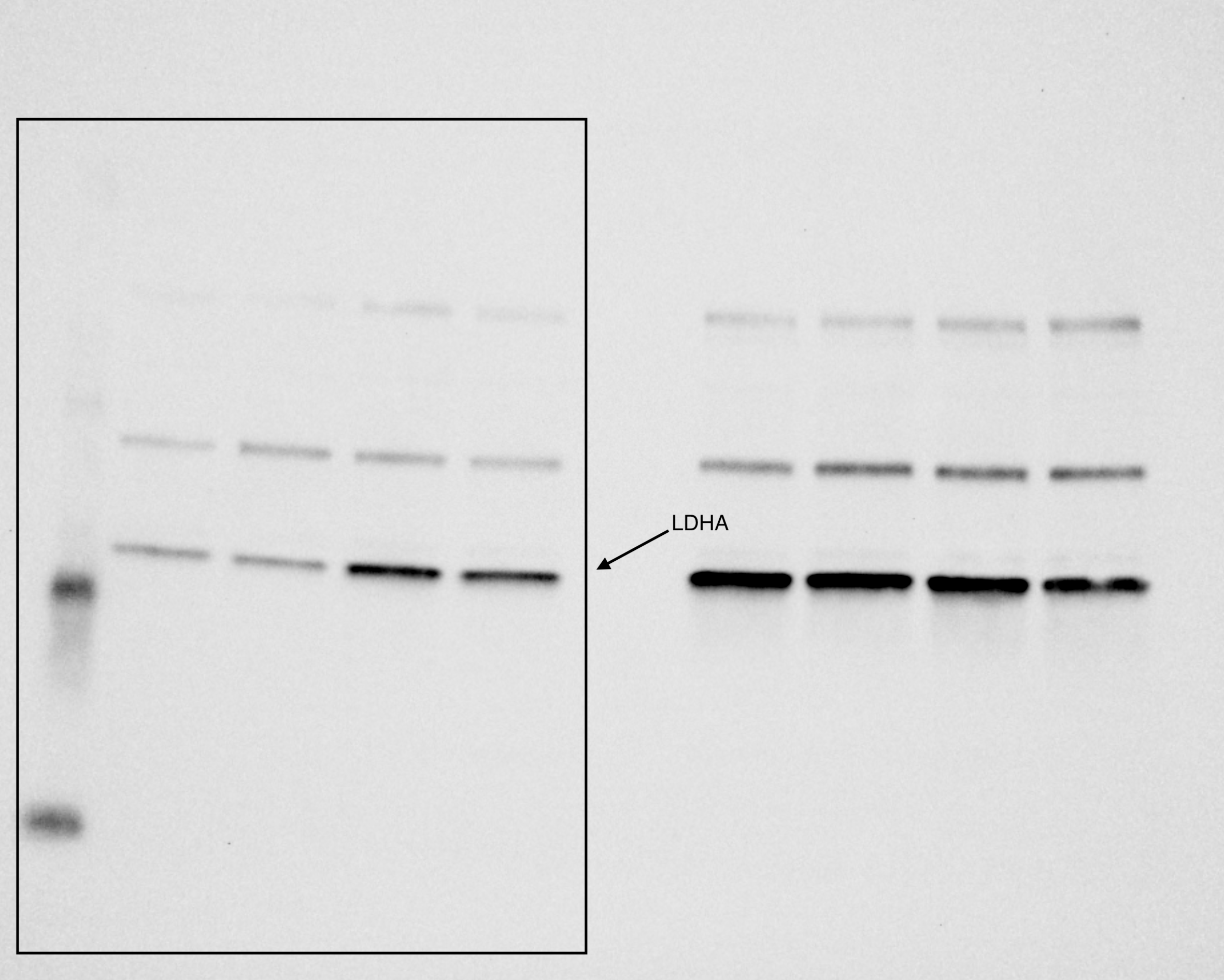

Supplement: Figure 1—source data 2. — Uncropped Western blot images of HK2, LDHA, and α-tubulin in TR-AMs treated with echinomycin under normoxia or hypoxia. [file elife-77457-fig1-data2.zip › Figure 1-source data 2 (Figure 1E)/Figure 1E-LDHA.tif]

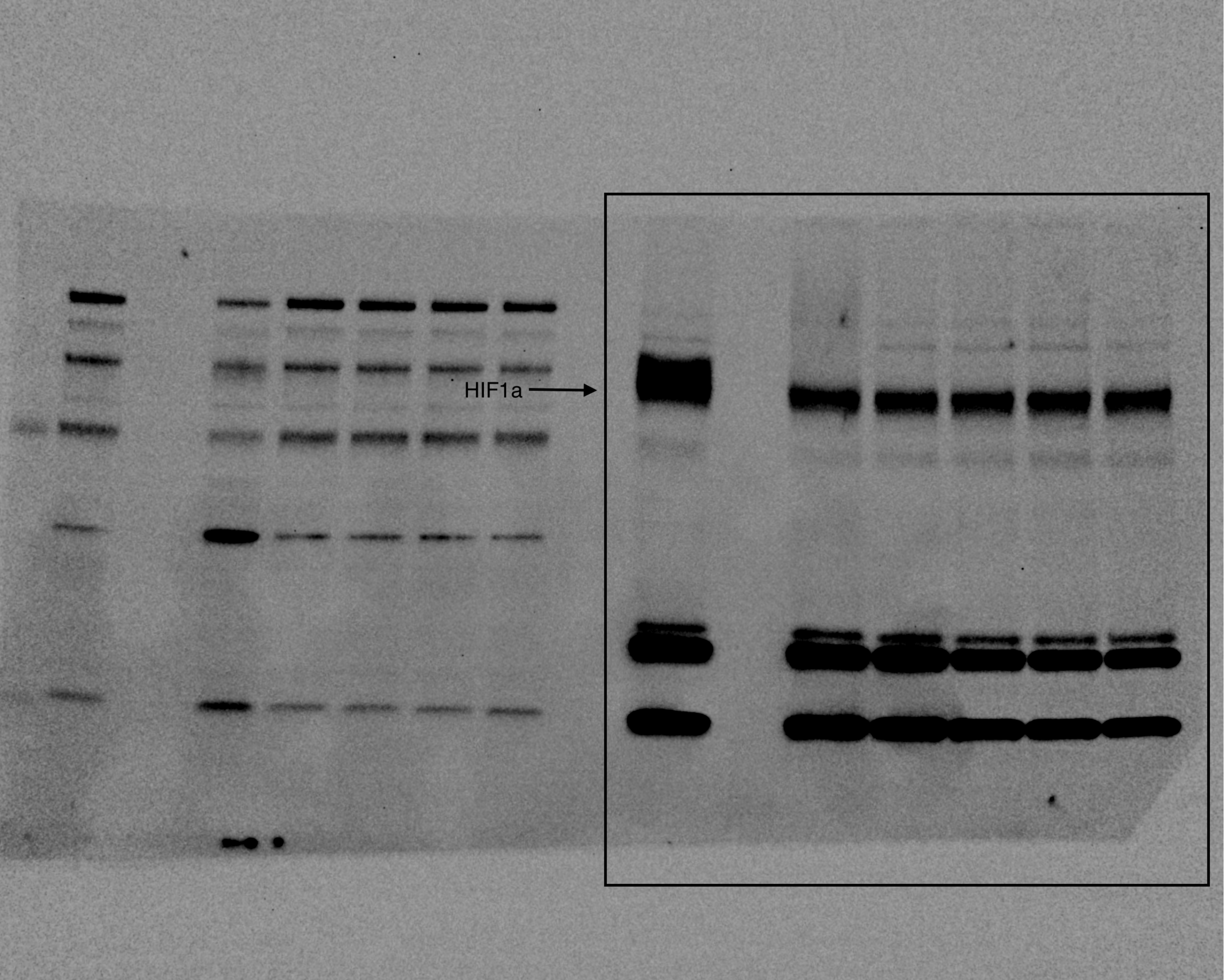

Supplement: Figure 1—source data 3. — Uncropped Western blot images of HIF-1α protein expression in BMDMs under different concentrations of oxygen. [file elife-77457-fig1-data3.zip › Figure 1-source data 3 (Figure 1H)/Figure 1H-HIF1a.tif]

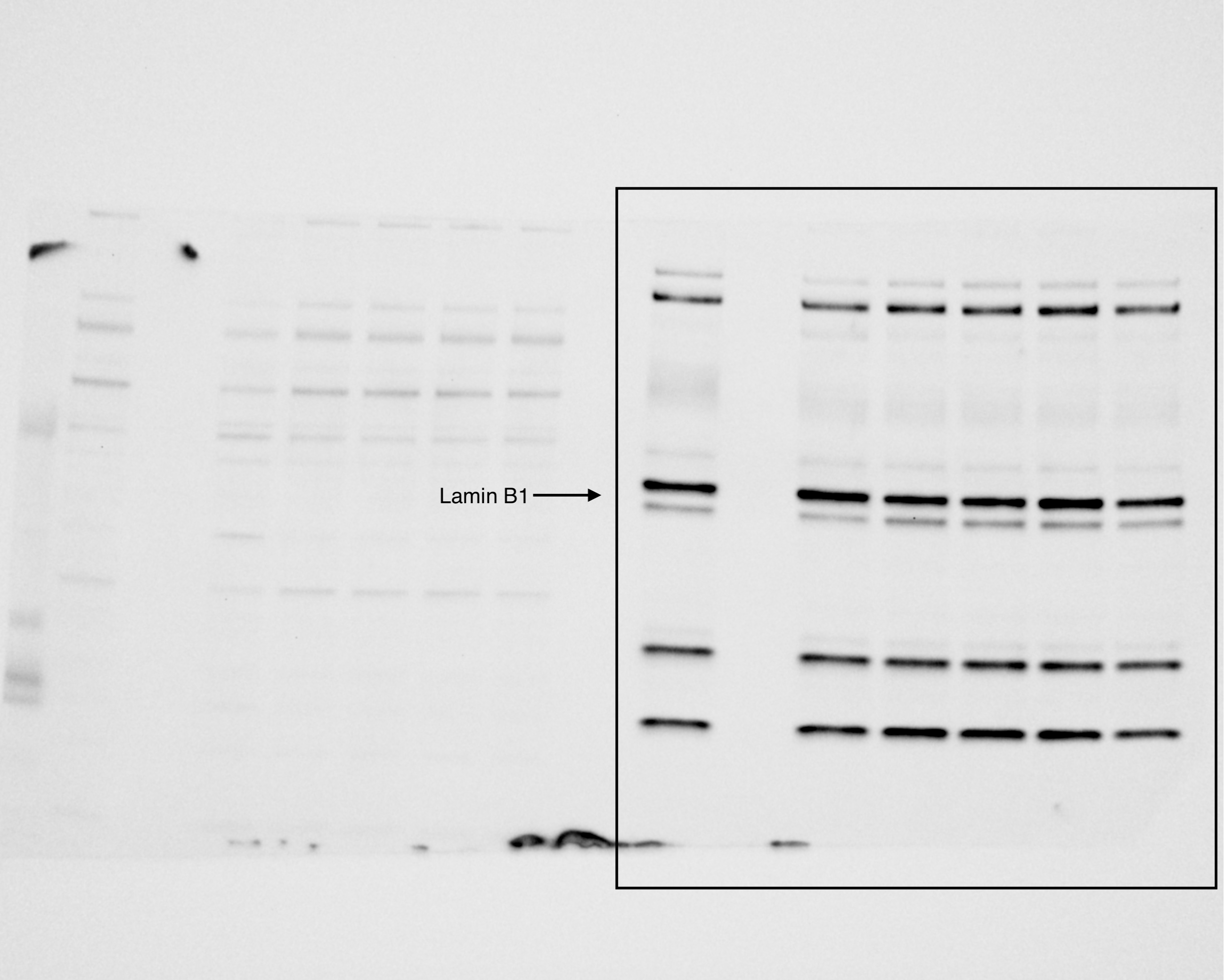

Supplement: Figure 1—source data 3. — Uncropped Western blot images of HIF-1α protein expression in BMDMs under different concentrations of oxygen. [file elife-77457-fig1-data3.zip › Figure 1-source data 3 (Figure 1H)/Figure 1H-LaminB1.tif]

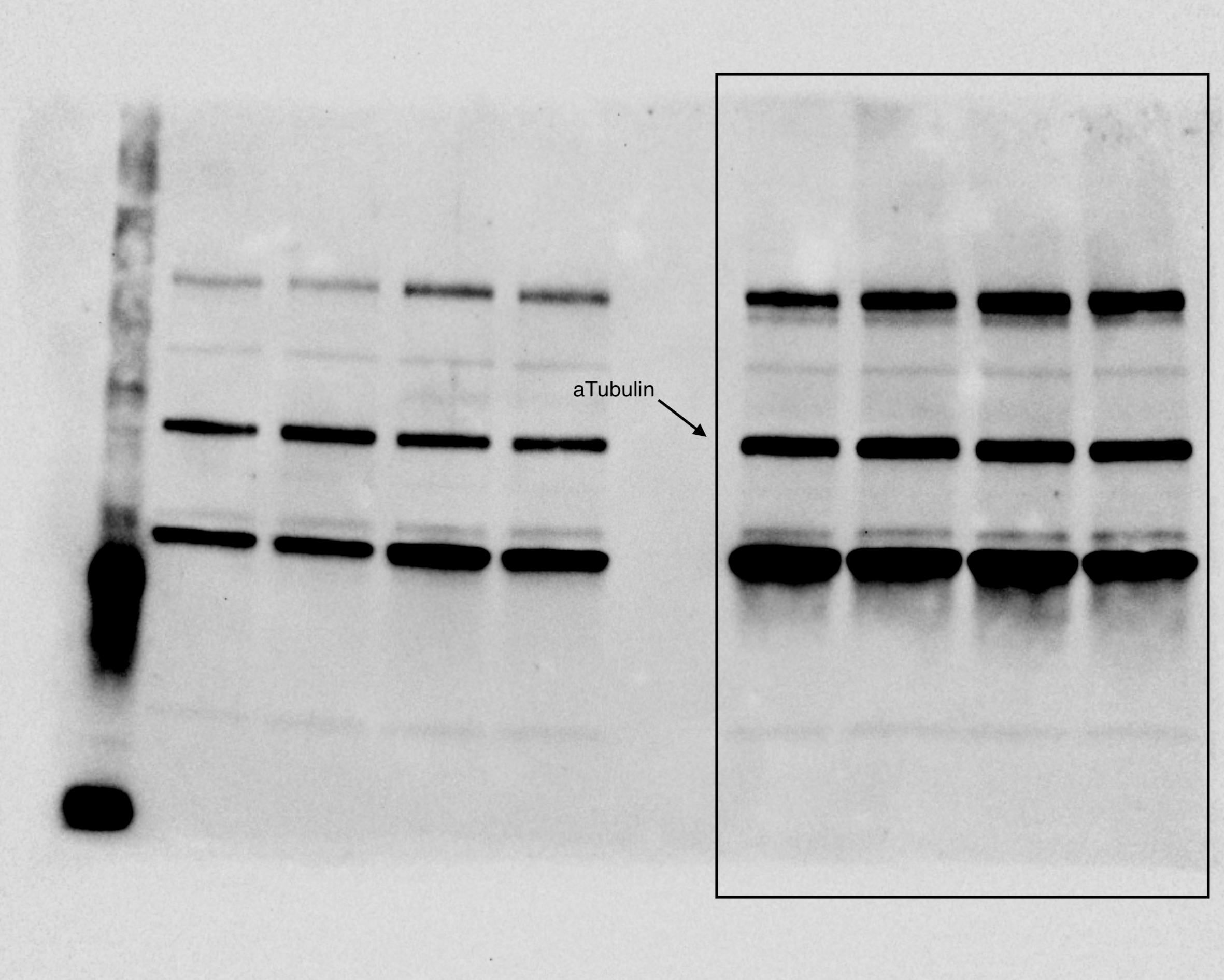

Supplement: Figure 1—source data 4. — Uncropped Western blot images of HK2, LDHA, and α-tubulin in BMDMs treated with echinomycin under normoxia or hypoxia. [file elife-77457-fig1-data4.zip › Figure 1-source data 4 (Figure 1J)/Figure 1J-aTubulin.tif]

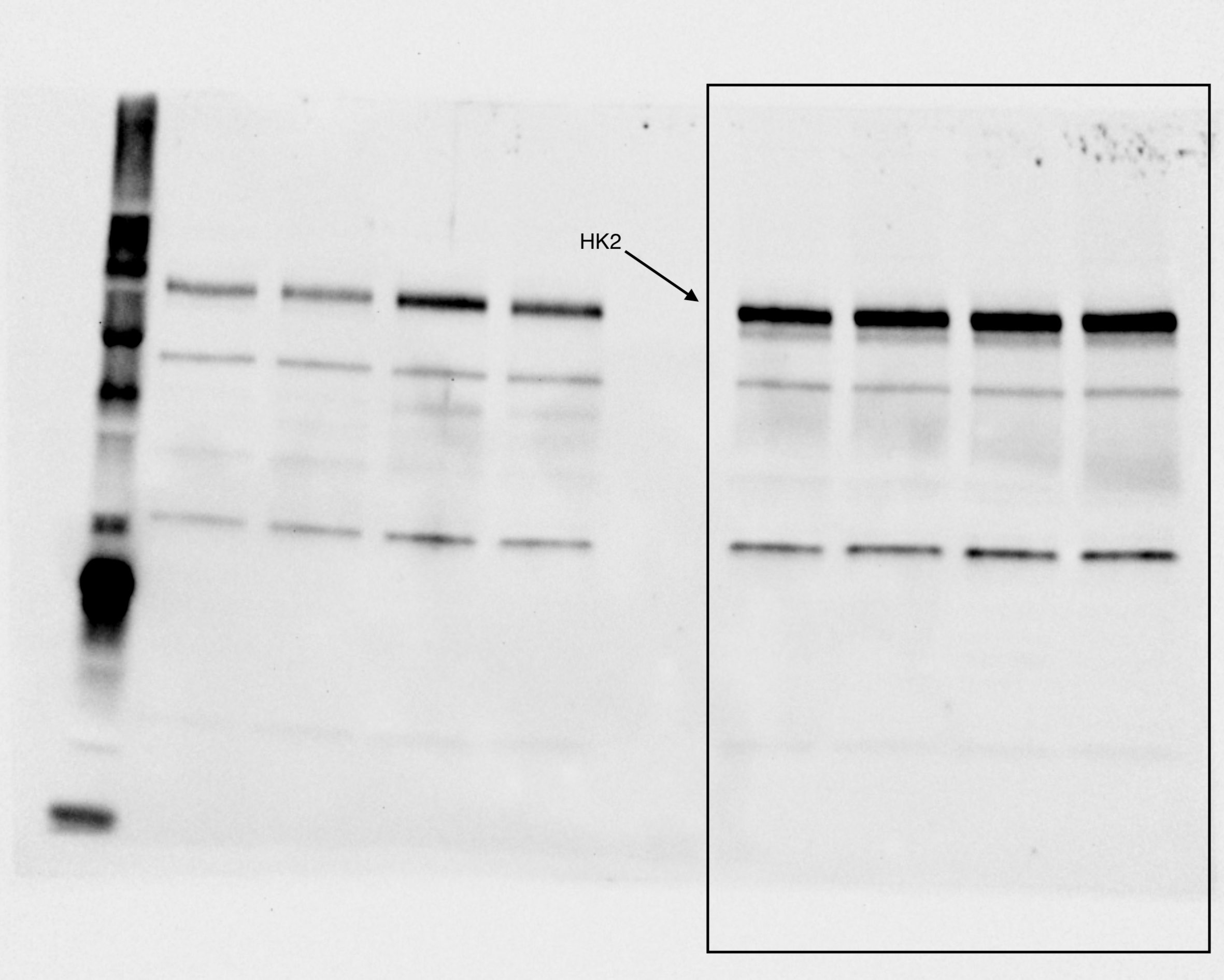

Supplement: Figure 1—source data 4. — Uncropped Western blot images of HK2, LDHA, and α-tubulin in BMDMs treated with echinomycin under normoxia or hypoxia. [file elife-77457-fig1-data4.zip › Figure 1-source data 4 (Figure 1J)/Figure 1J-HK2.tif]

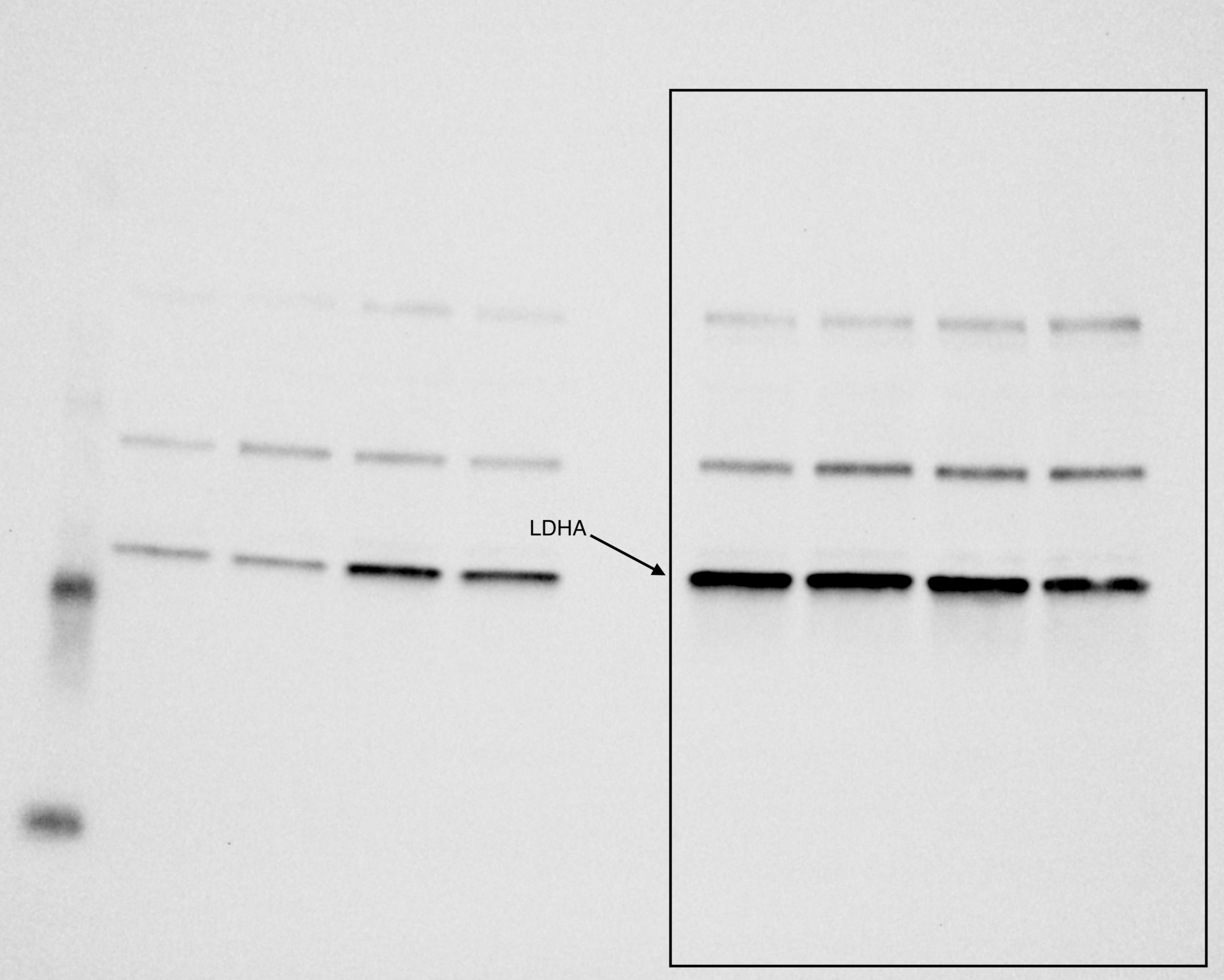

Supplement: Figure 1—source data 4. — Uncropped Western blot images of HK2, LDHA, and α-tubulin in BMDMs treated with echinomycin under normoxia or hypoxia. [file elife-77457-fig1-data4.zip › Figure 1-source data 4 (Figure 1J)/Figure 1J-LDHA.tif]

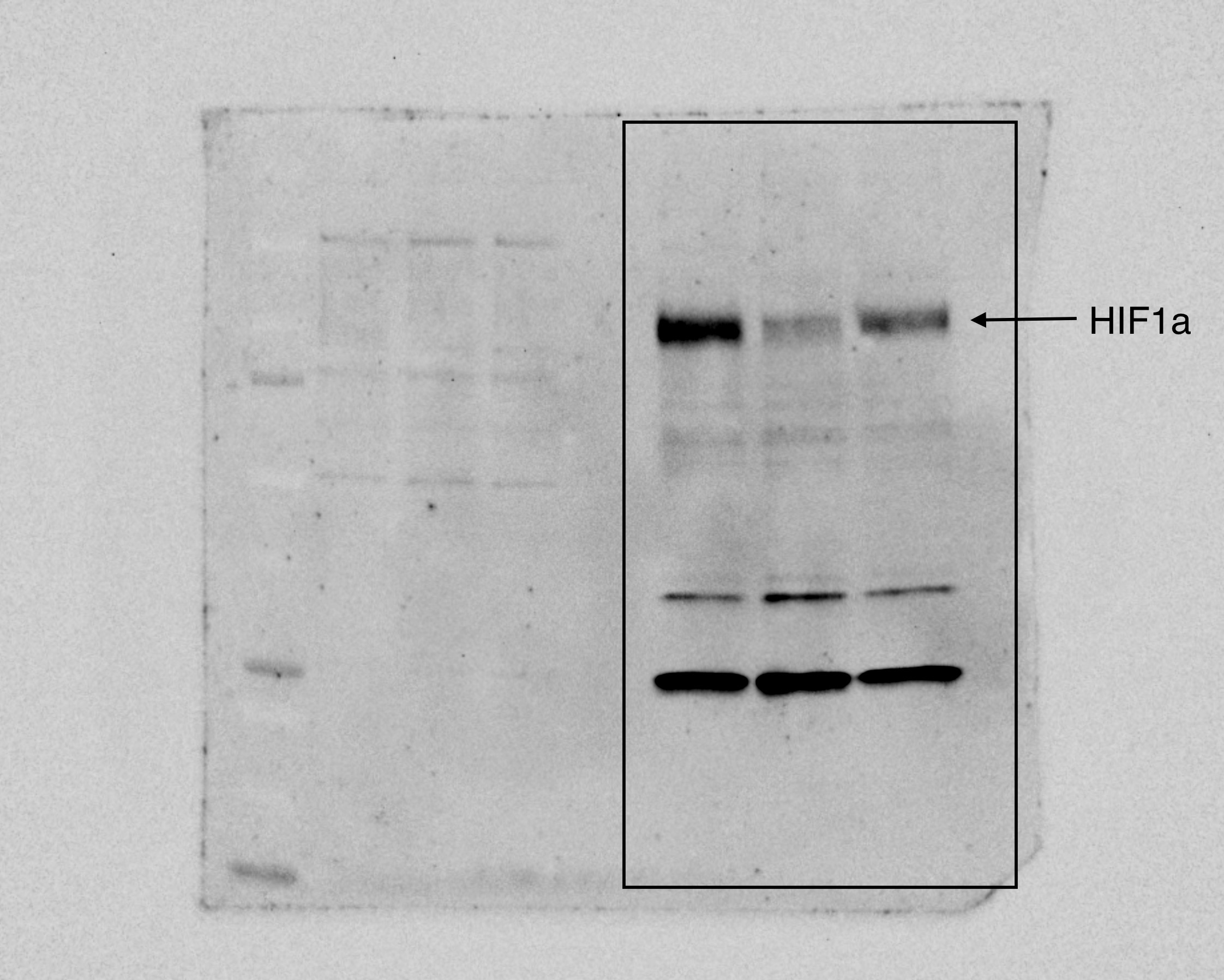

Supplement: Figure 1—figure supplement 1—source data 1. — Uncropped Western blot images of hypoxia-inducible factor 1-alpha (HIF-1α) protein expression in TR-AMs treated with either control siRNA or two different Hif1a siRNAs. [file elife-77457-fig1-figsupp1-data1.zip › Figure 1-figure supplement 1-source data 1 (Figure supplement 1A)/Fig 1-fig suppl 1A-HIF1a.tiff]

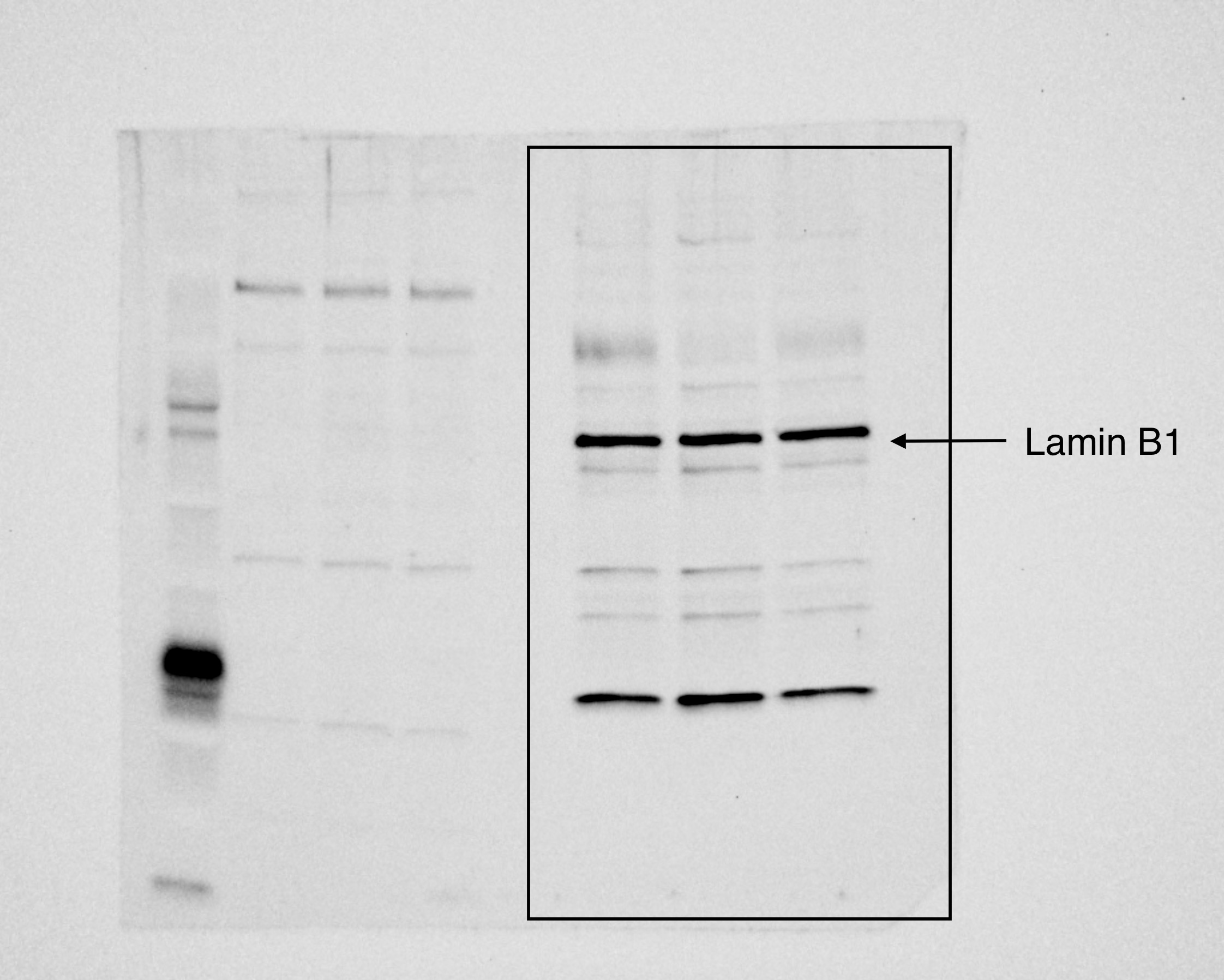

Supplement: Figure 1—figure supplement 1—source data 1. — Uncropped Western blot images of hypoxia-inducible factor 1-alpha (HIF-1α) protein expression in TR-AMs treated with either control siRNA or two different Hif1a siRNAs. [file elife-77457-fig1-figsupp1-data1.zip › Figure 1-figure supplement 1-source data 1 (Figure supplement 1A)/Fig 1-fig suppl 1A-LaminB1.tiff]

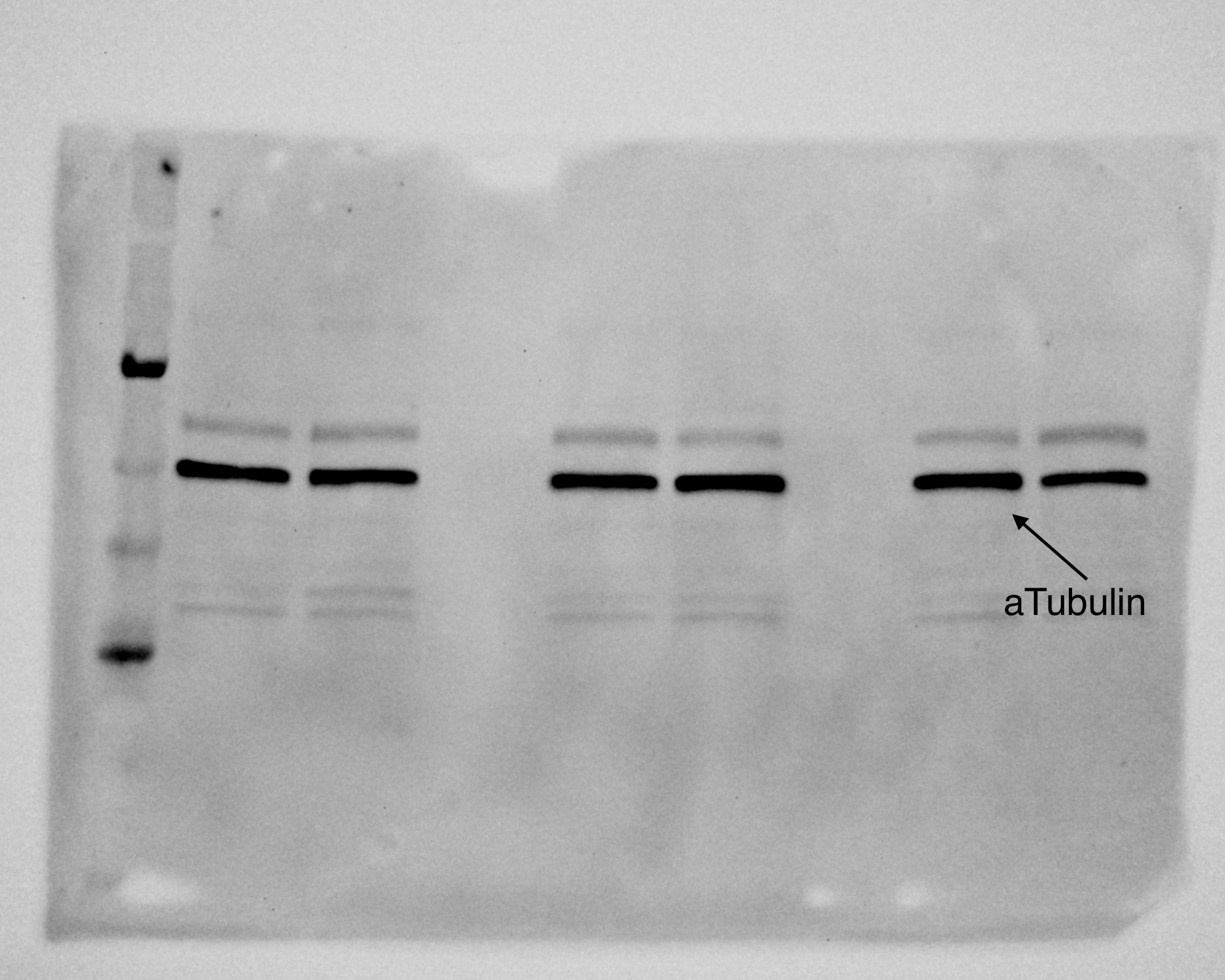

Supplement: Figure 1—figure supplement 1—source data 2. — Uncropped Western blot images of HK2, LDHA, and α-tubulin expression in TR-AMs treated with either control siRNA or two different Hif1a siRNAs under normoxia or hypoxia. [file elife-77457-fig1-figsupp1-data2.zip › Figure 1-figure supplement 1-source data 2 (Figure supplement 1B)/Fig 1-fig suppl 1B-aTubulin.tiff]

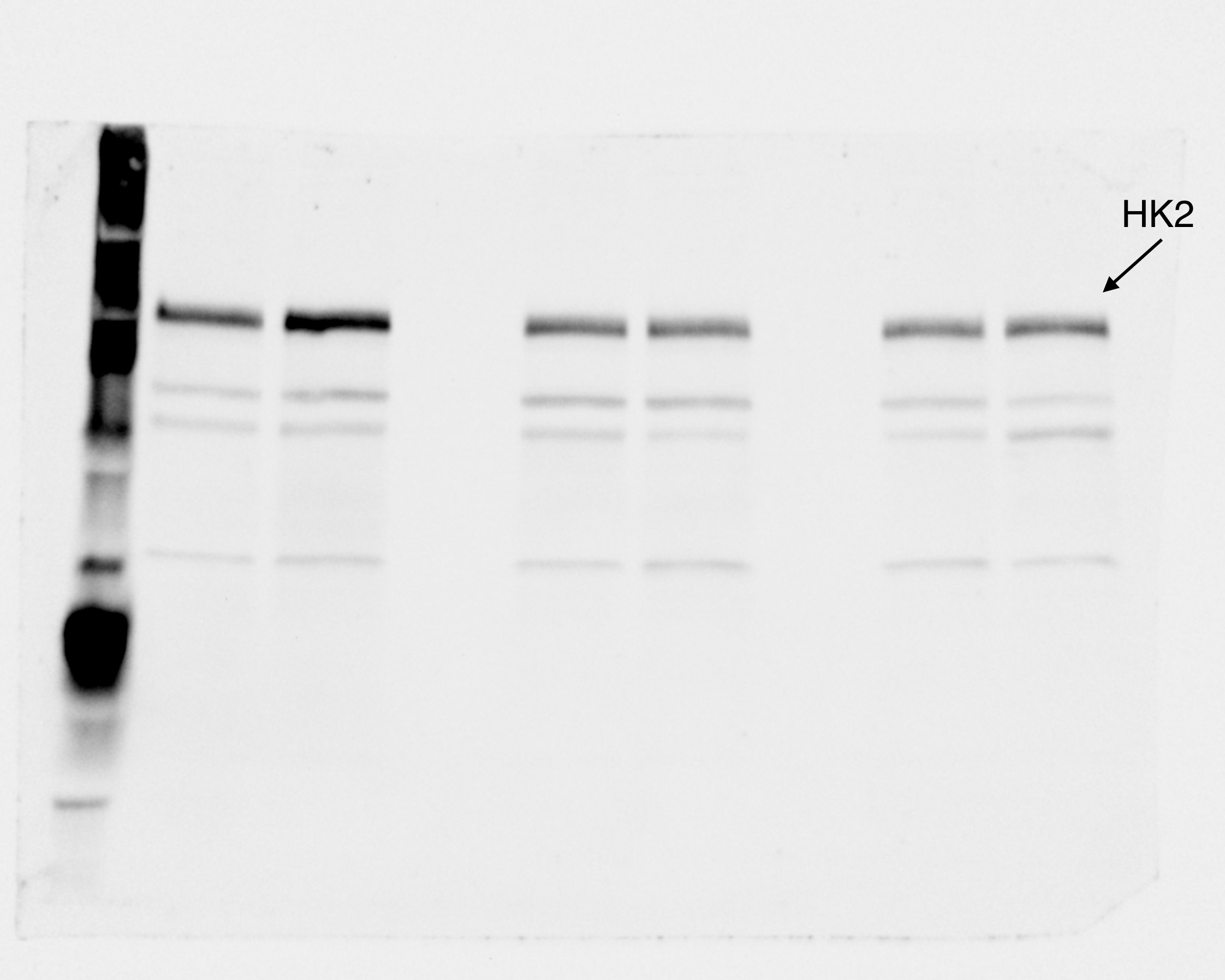

Supplement: Figure 1—figure supplement 1—source data 2. — Uncropped Western blot images of HK2, LDHA, and α-tubulin expression in TR-AMs treated with either control siRNA or two different Hif1a siRNAs under normoxia or hypoxia. [file elife-77457-fig1-figsupp1-data2.zip › Figure 1-figure supplement 1-source data 2 (Figure supplement 1B)/Fig 1-fig suppl 1B-HK2.tiff]

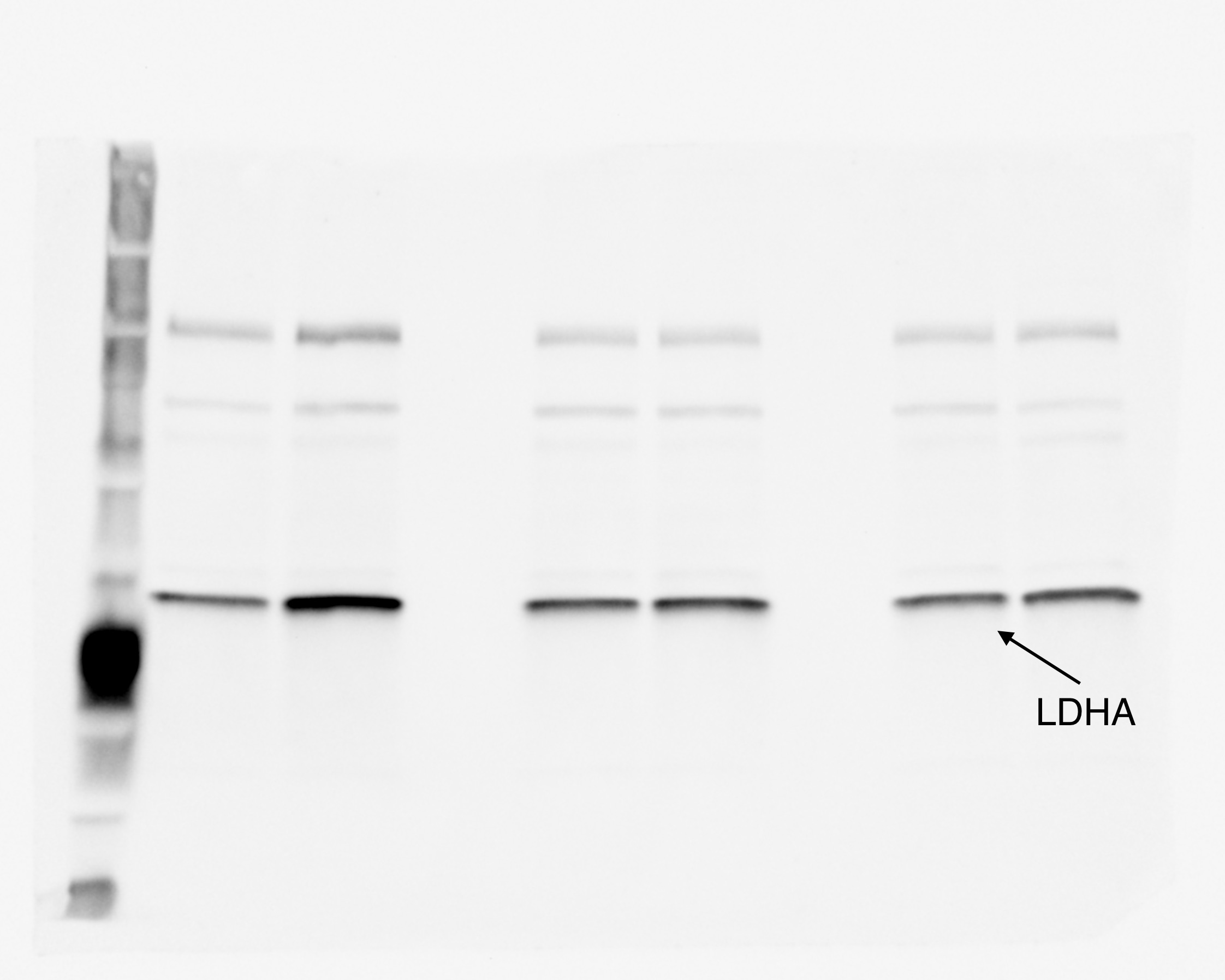

Supplement: Figure 1—figure supplement 1—source data 2. — Uncropped Western blot images of HK2, LDHA, and α-tubulin expression in TR-AMs treated with either control siRNA or two different Hif1a siRNAs under normoxia or hypoxia. [file elife-77457-fig1-figsupp1-data2.zip › Figure 1-figure supplement 1-source data 2 (Figure supplement 1B)/Fig 1-fig suppl 1B-LDHA.tiff]

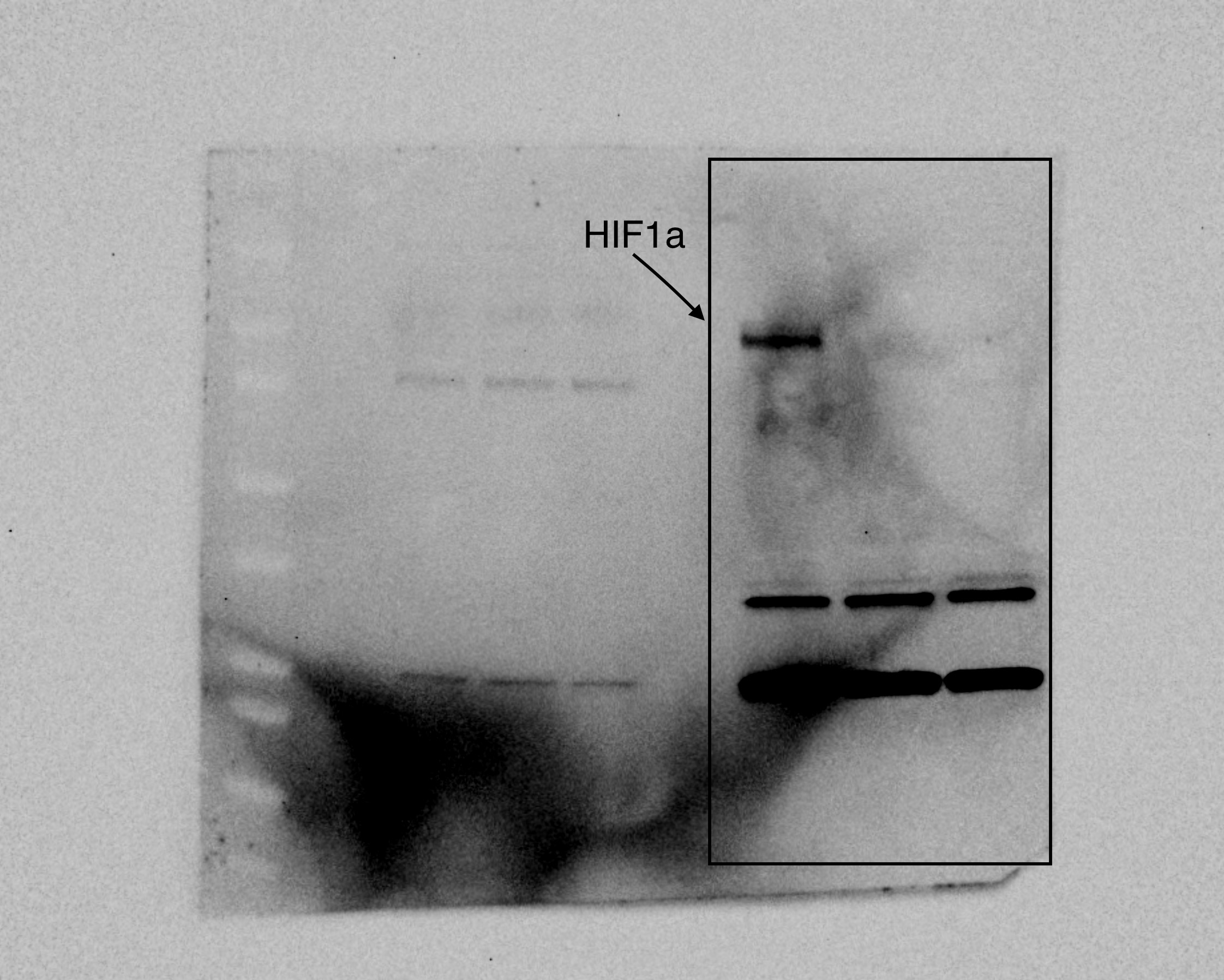

Supplement: Figure 1—figure supplement 1—source data 3. — Uncropped Western blot images of hypoxia-inducible factor 1-alpha (HIF-1α) protein expression in BMDMs treated with either control siRNA or two different Hif1a siRNAs. [file elife-77457-fig1-figsupp1-data3.zip › Figure 1-figure supplement 1-source data 3 (Figure supplement 1D)/Fig 1-fig suppl 1D-HIF1a.tiff]

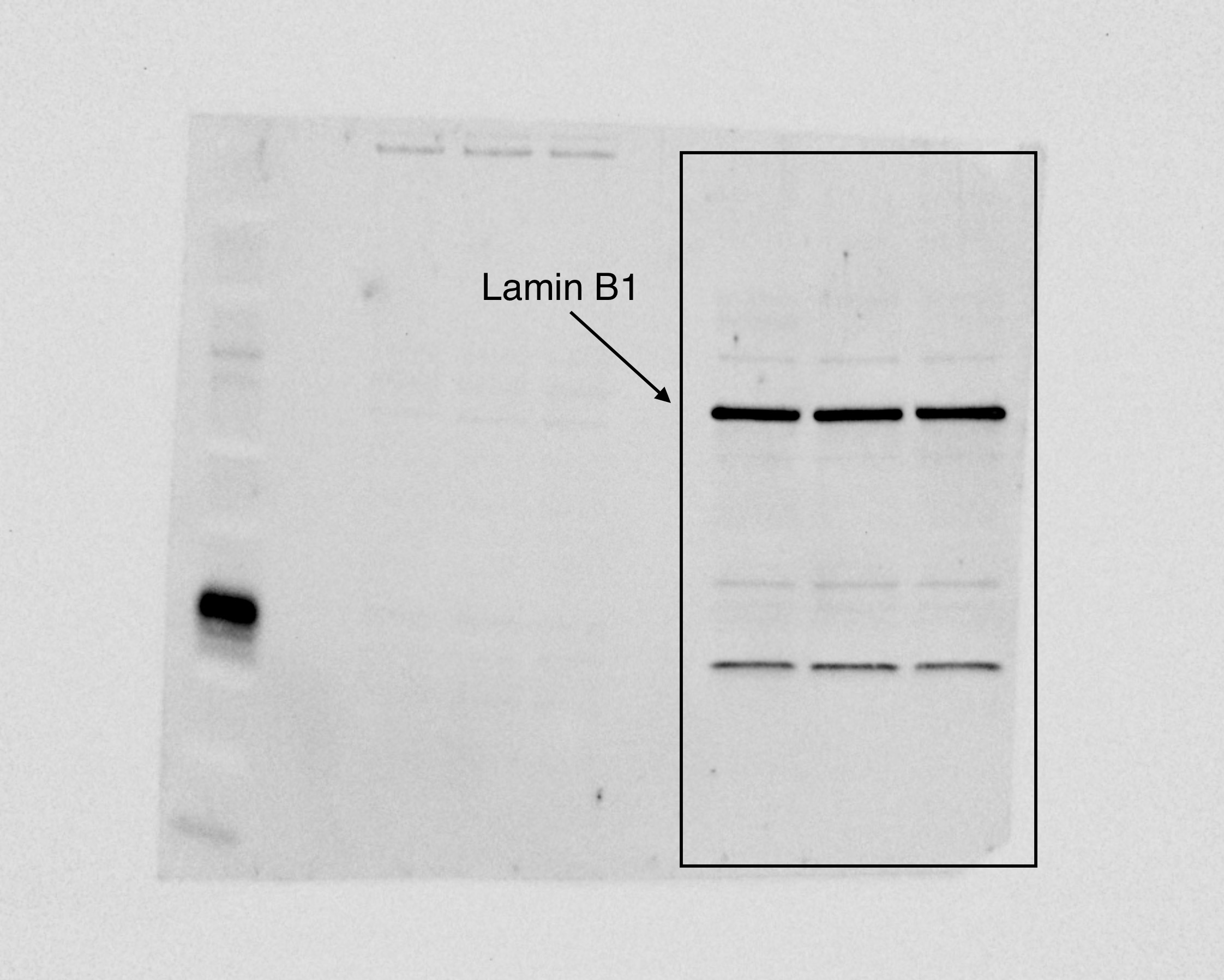

Supplement: Figure 1—figure supplement 1—source data 3. — Uncropped Western blot images of hypoxia-inducible factor 1-alpha (HIF-1α) protein expression in BMDMs treated with either control siRNA or two different Hif1a siRNAs. [file elife-77457-fig1-figsupp1-data3.zip › Figure 1-figure supplement 1-source data 3 (Figure supplement 1D)/Fig1-fig suppl 1D-LaminB1.tiff]

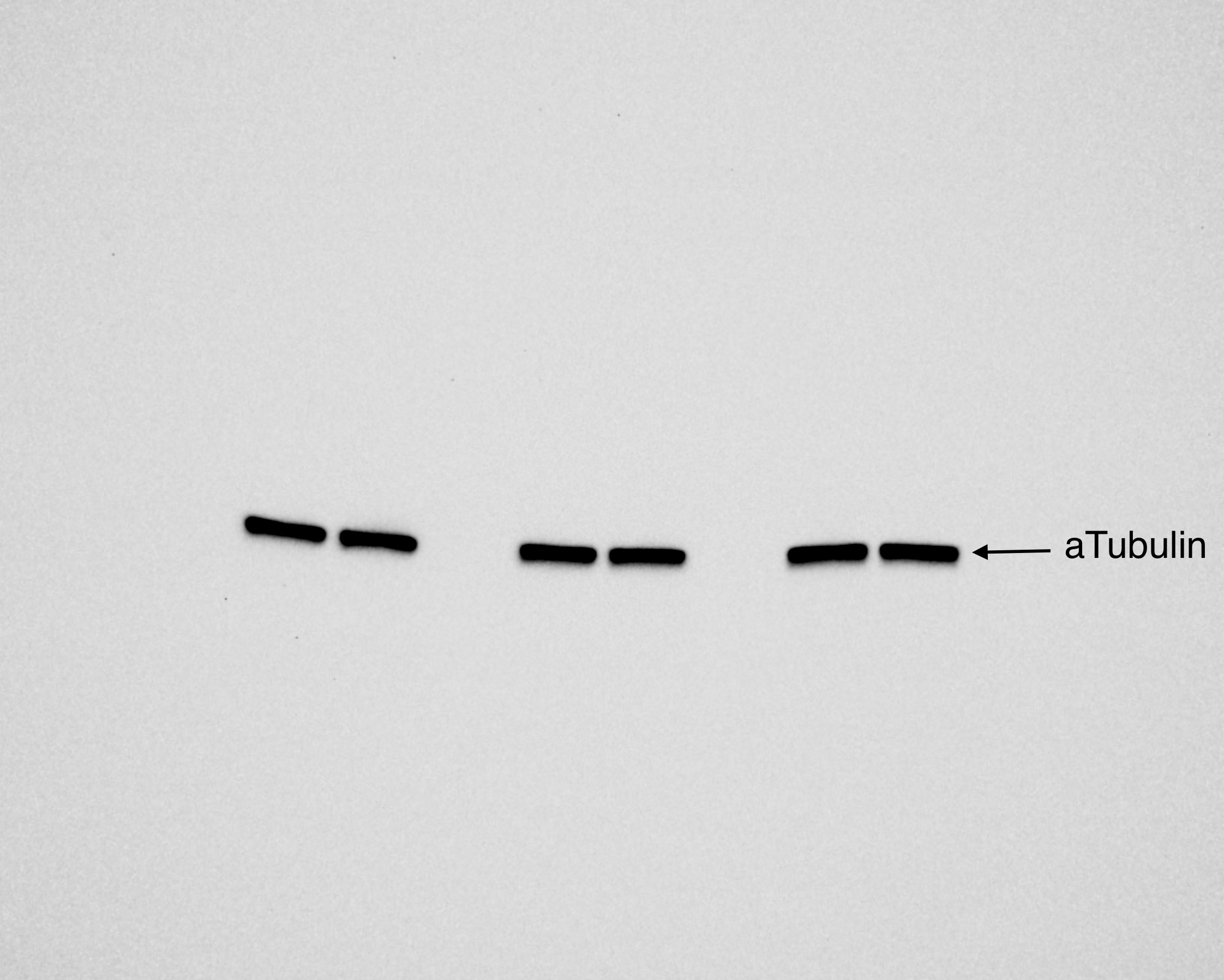

Supplement: Figure 1—figure supplement 1—source data 4. — Uncropped Western blot images of HK2, LDHA, and α-tubulin expression in BMDMs treated with either control siRNA or two different Hif1a siRNAs under normoxia or hypoxia. [file elife-77457-fig1-figsupp1-data4.zip › Figure 1-figure supplement 1-source data 4 (Figure supplement 1E)/Fig 1-fig suppl 1E-aTubulin.tiff]

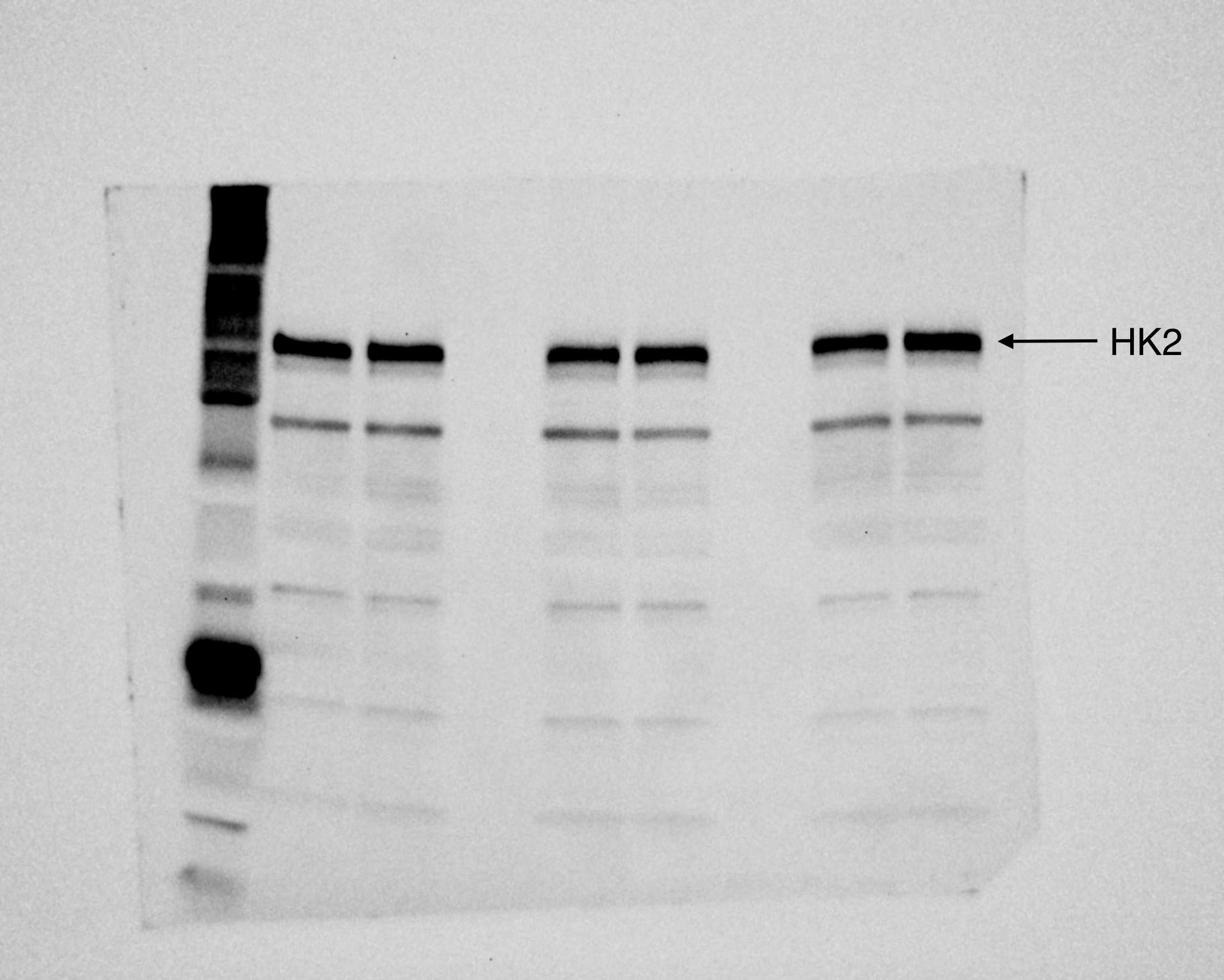

Supplement: Figure 1—figure supplement 1—source data 4. — Uncropped Western blot images of HK2, LDHA, and α-tubulin expression in BMDMs treated with either control siRNA or two different Hif1a siRNAs under normoxia or hypoxia. [file elife-77457-fig1-figsupp1-data4.zip › Figure 1-figure supplement 1-source data 4 (Figure supplement 1E)/Fig 1-fig suppl 1E-HK2.tiff]

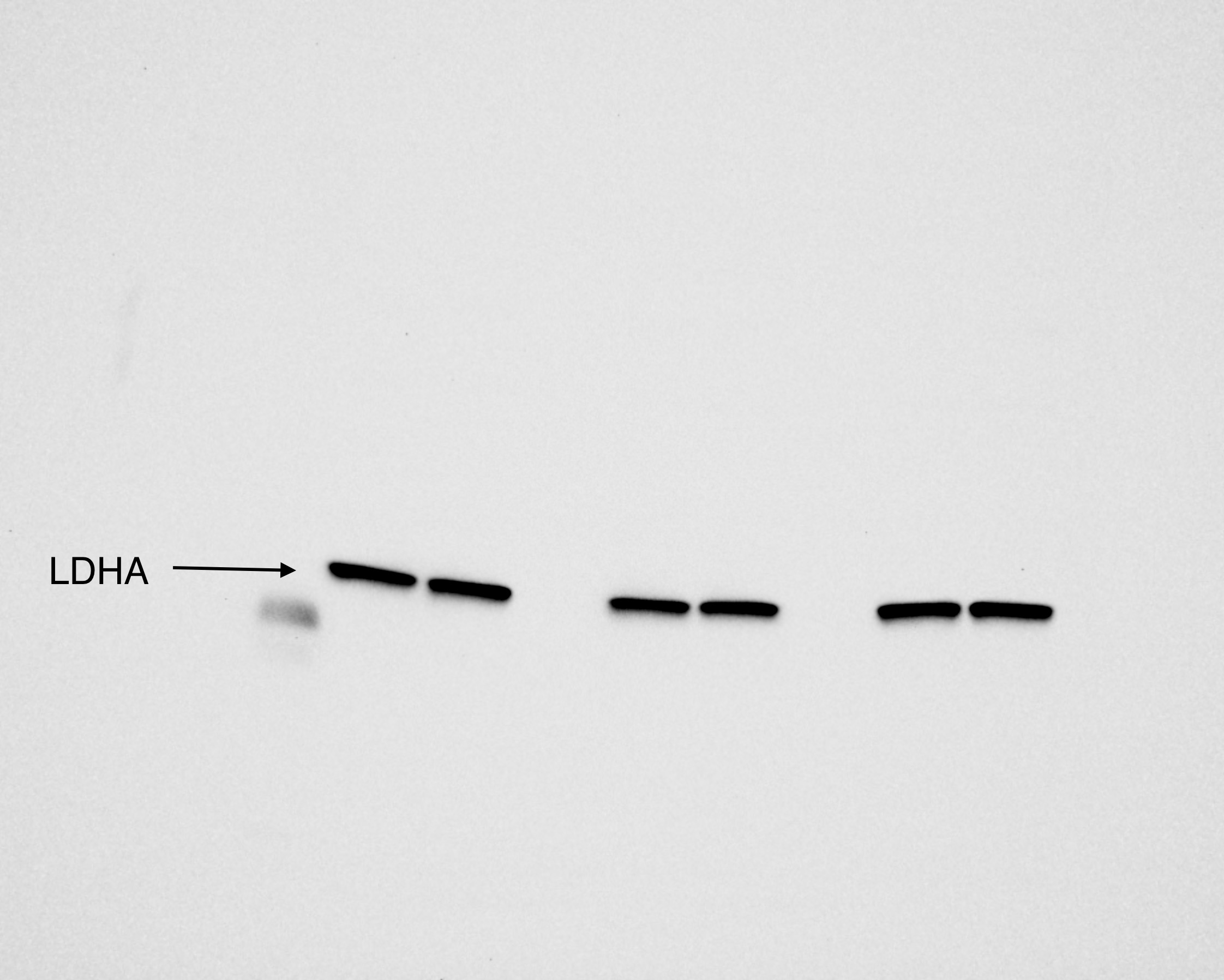

Supplement: Figure 1—figure supplement 1—source data 4. — Uncropped Western blot images of HK2, LDHA, and α-tubulin expression in BMDMs treated with either control siRNA or two different Hif1a siRNAs under normoxia or hypoxia. [file elife-77457-fig1-figsupp1-data4.zip › Figure 1-figure supplement 1-source data 4 (Figure supplement 1E)/Fig 1-fig suppl 1E-LDHA.tiff]

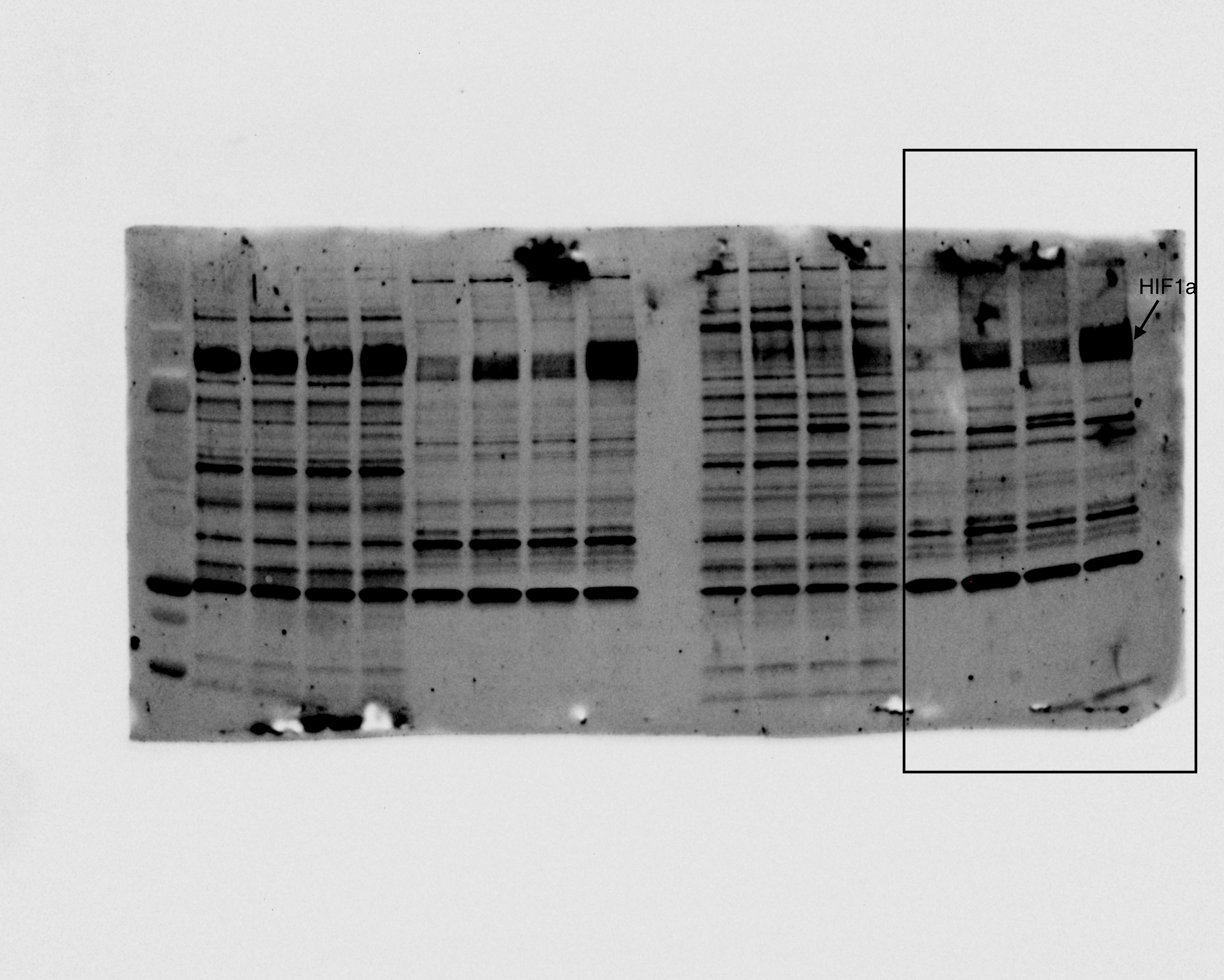

Supplement: Figure 1—figure supplement 2—source data 1. — Uncropped Western blot images of HIF-1α protein expression in TR-AMs treated with hypoxia for 0, 2, or 16 hr or with DMOG. [file elife-77457-fig1-figsupp2-data1.zip › Figure 1-figure supplement 2-source data 1 (Figure supplement 2A)/Fig 1-fig suppl 2A-Hif1a.tif]

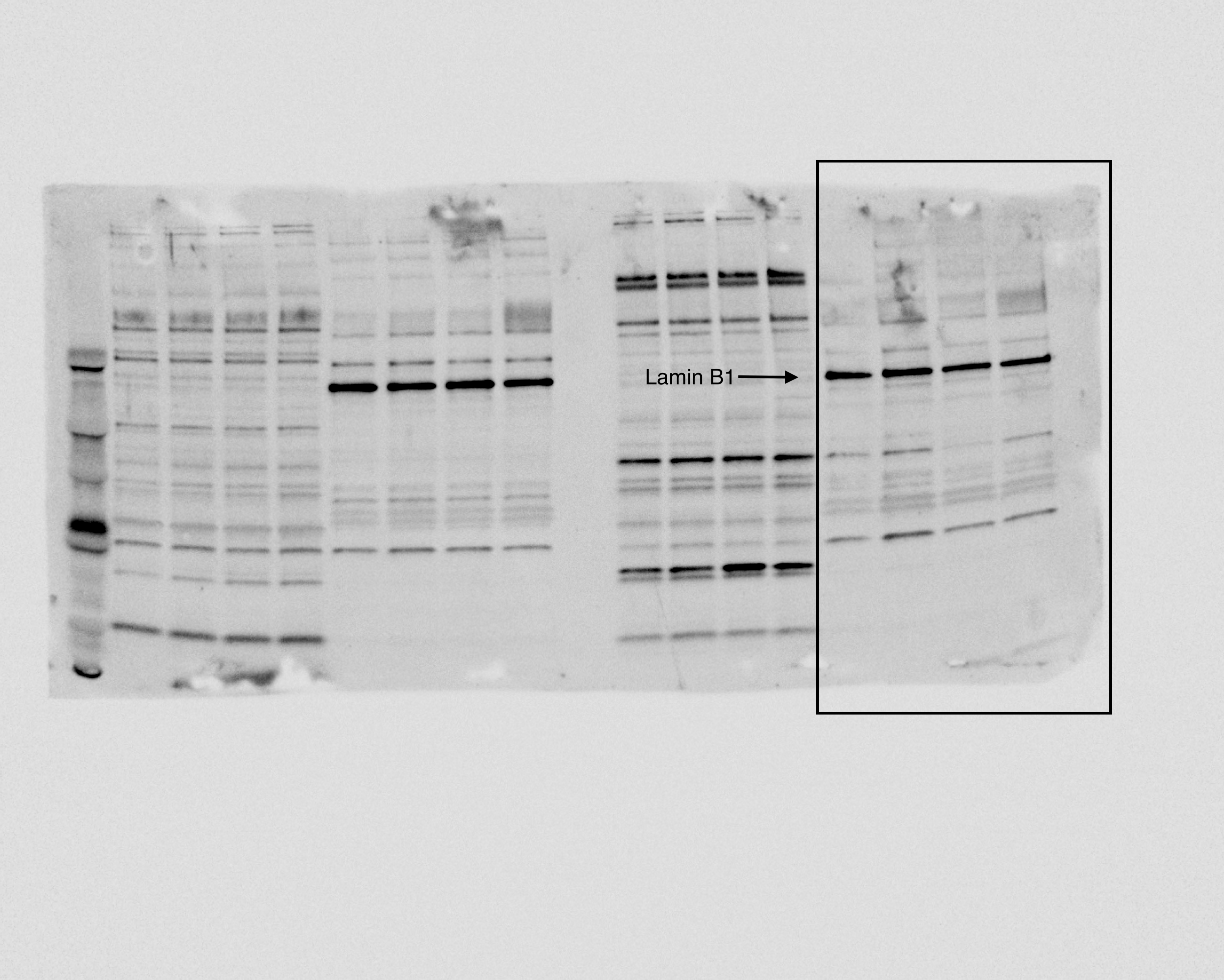

Supplement: Figure 1—figure supplement 2—source data 1. — Uncropped Western blot images of HIF-1α protein expression in TR-AMs treated with hypoxia for 0, 2, or 16 hr or with DMOG. [file elife-77457-fig1-figsupp2-data1.zip › Figure 1-figure supplement 2-source data 1 (Figure supplement 2A)/Fig 1-fig suppl 2A-LaminB1.tif]

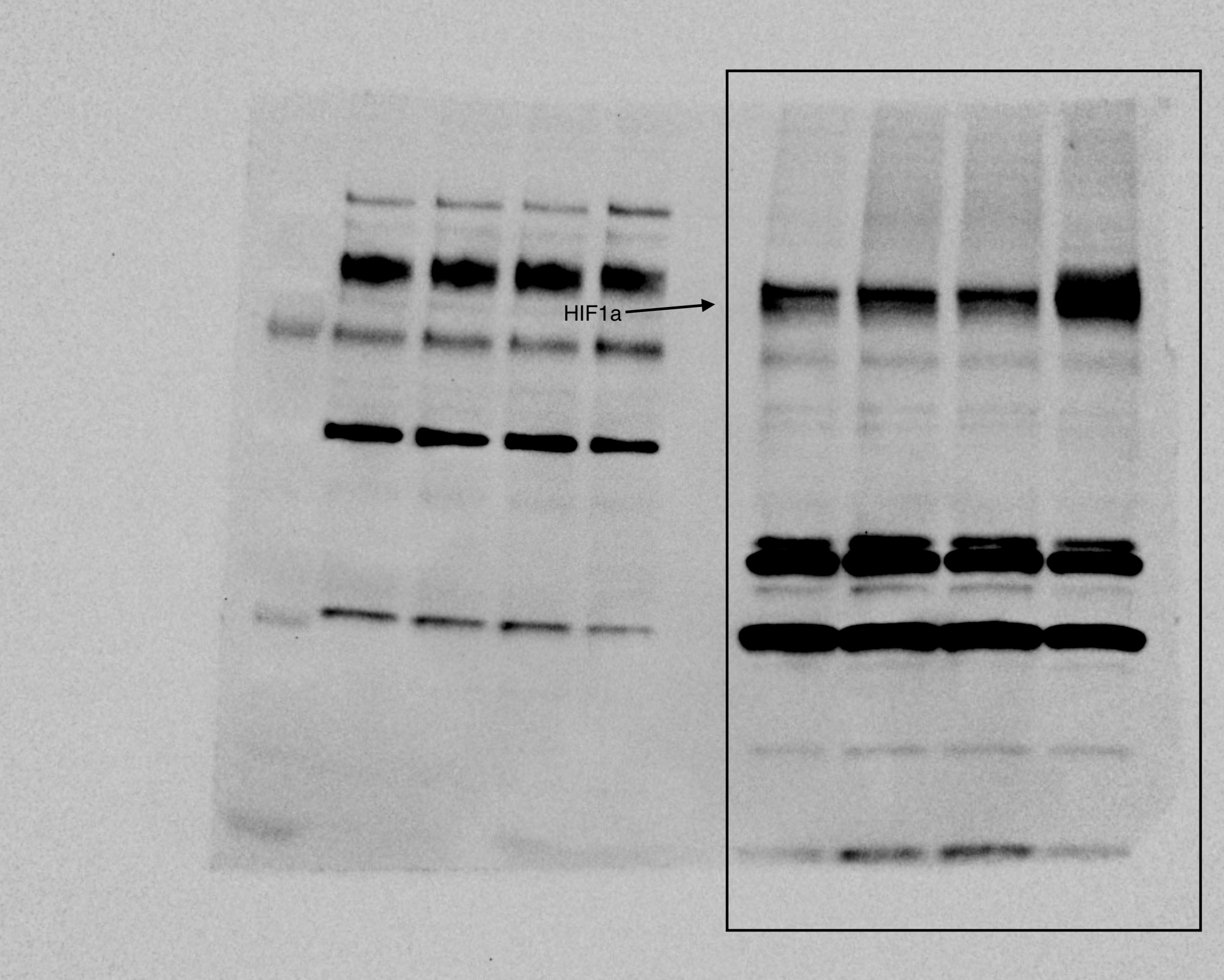

Supplement: Figure 1—figure supplement 2—source data 2. — Uncropped Western blot images of HIF-1α protein expression in BMDMs treated with hypoxia for 0, 2, or 16 hr or with DMOG. [file elife-77457-fig1-figsupp2-data2.zip › Figure 1-figure supplement 2-source data 2 (Figure supplement 2D)/Fig 1-fig suppl 2D-HIF1a.tif]

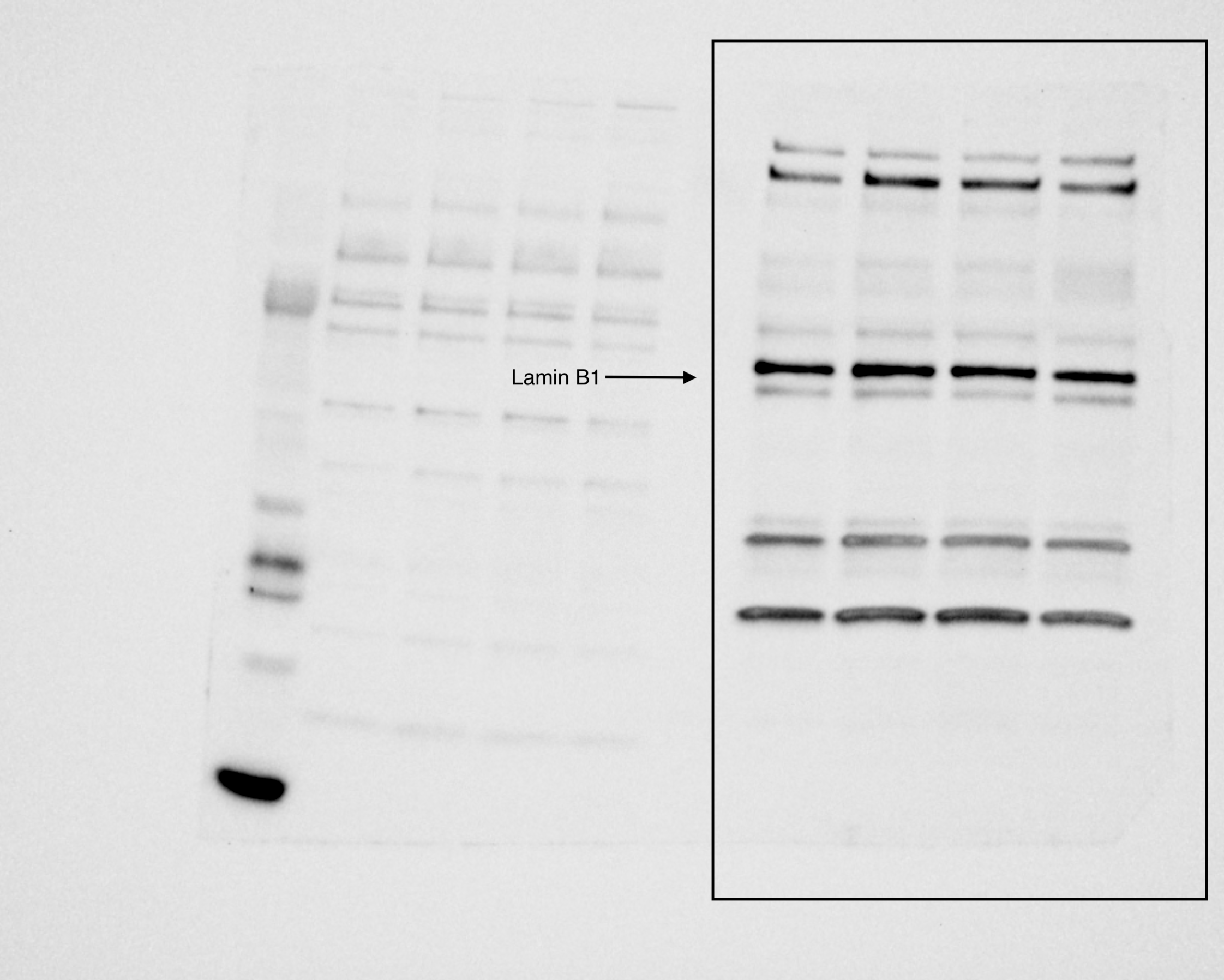

Supplement: Figure 1—figure supplement 2—source data 2. — Uncropped Western blot images of HIF-1α protein expression in BMDMs treated with hypoxia for 0, 2, or 16 hr or with DMOG. [file elife-77457-fig1-figsupp2-data2.zip › Figure 1-figure supplement 2-source data 2 (Figure supplement 2D)/Fig 1-fig suppl 2D-LaminB1.tif]

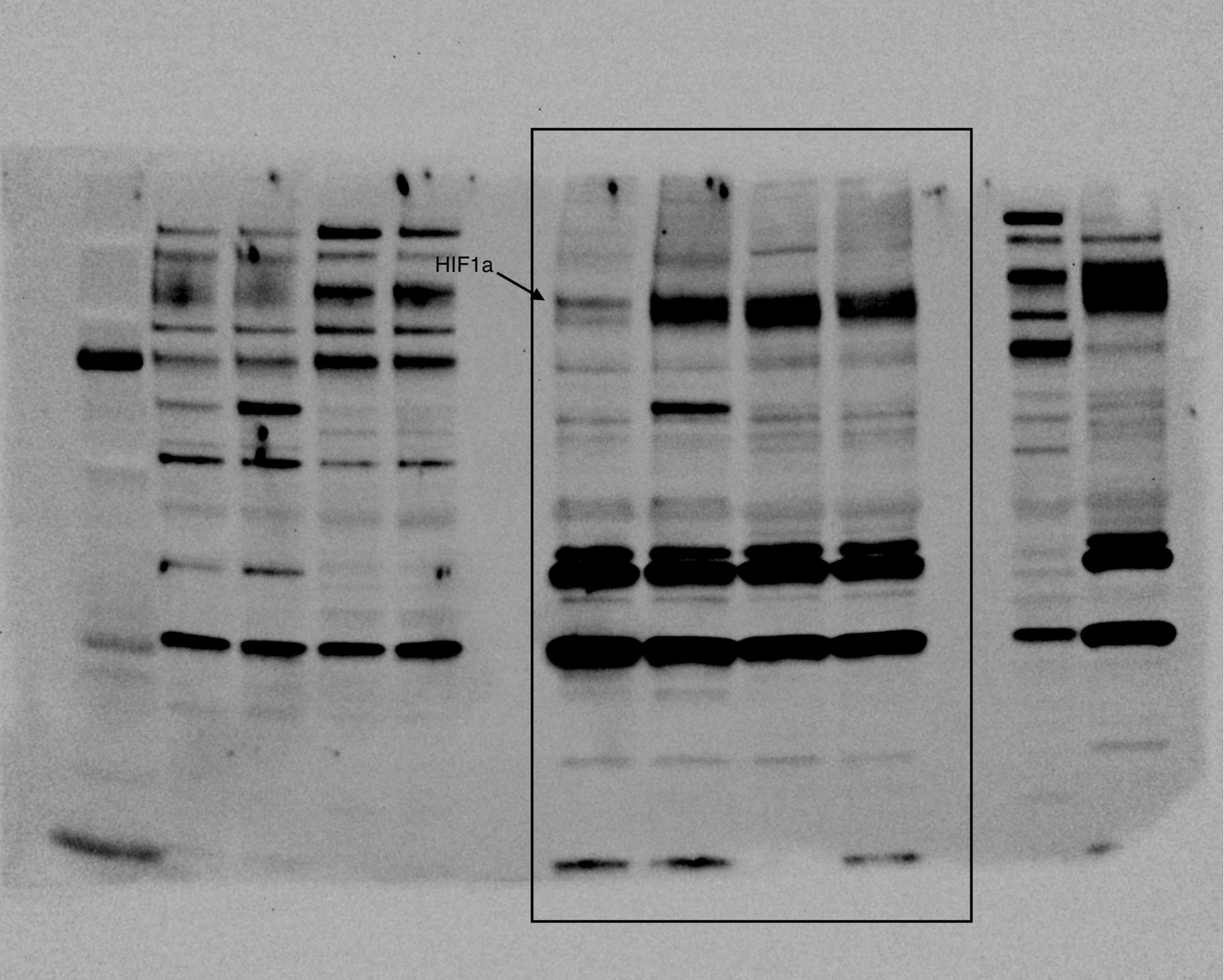

Supplement: Figure 2—source data 2. — Uncropped Western blot images of HIF-1α expression in TR-AMs and BMDMs treated with normoxia or hypoxia. [file elife-77457-fig2-data2.zip › Figure 2-source data 2 (Figure 2D)/Figure 2D-HIF1a.tif]

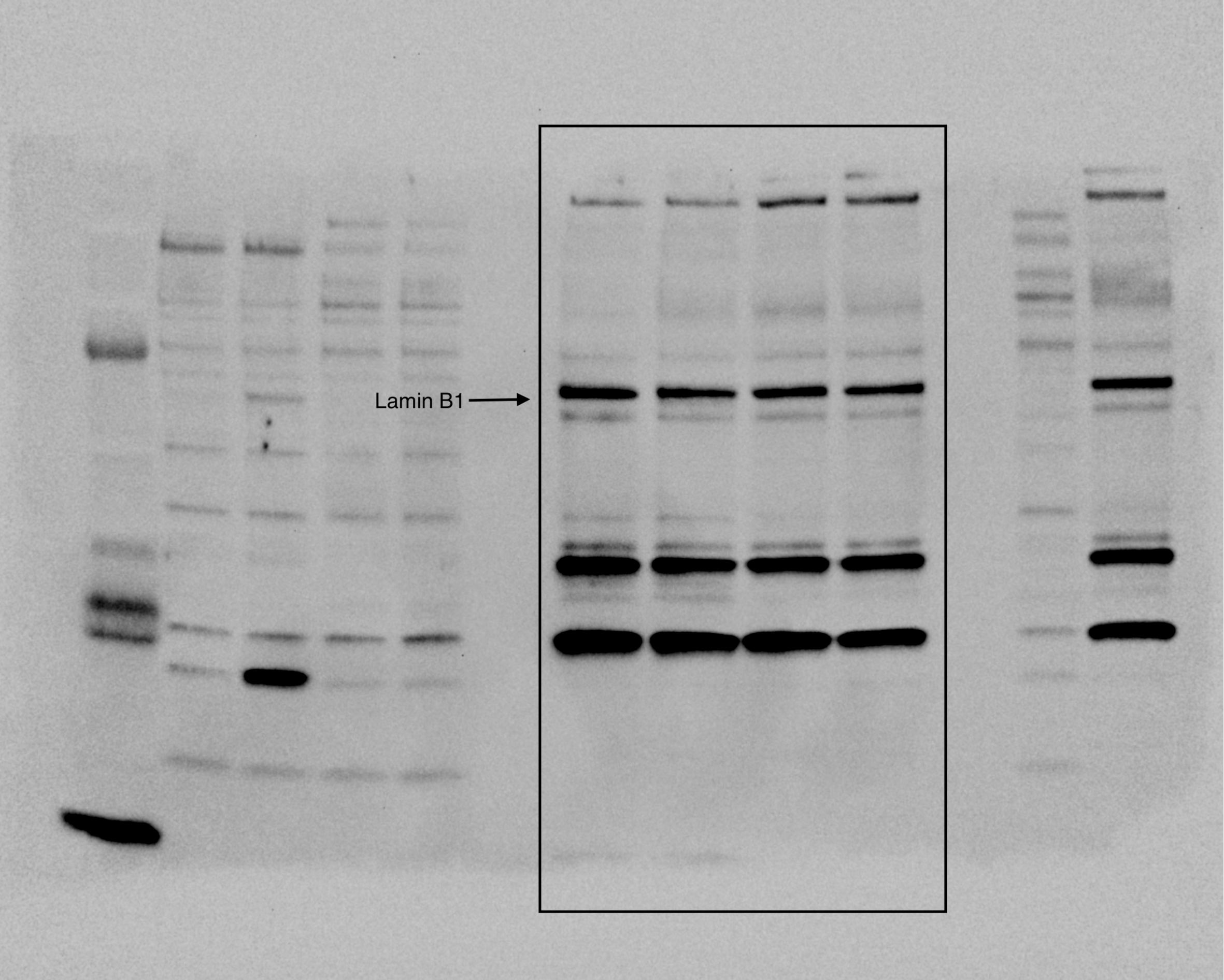

Supplement: Figure 2—source data 2. — Uncropped Western blot images of HIF-1α expression in TR-AMs and BMDMs treated with normoxia or hypoxia. [file elife-77457-fig2-data2.zip › Figure 2-source data 2 (Figure 2D)/Figure 2D-LaminB1.tif]

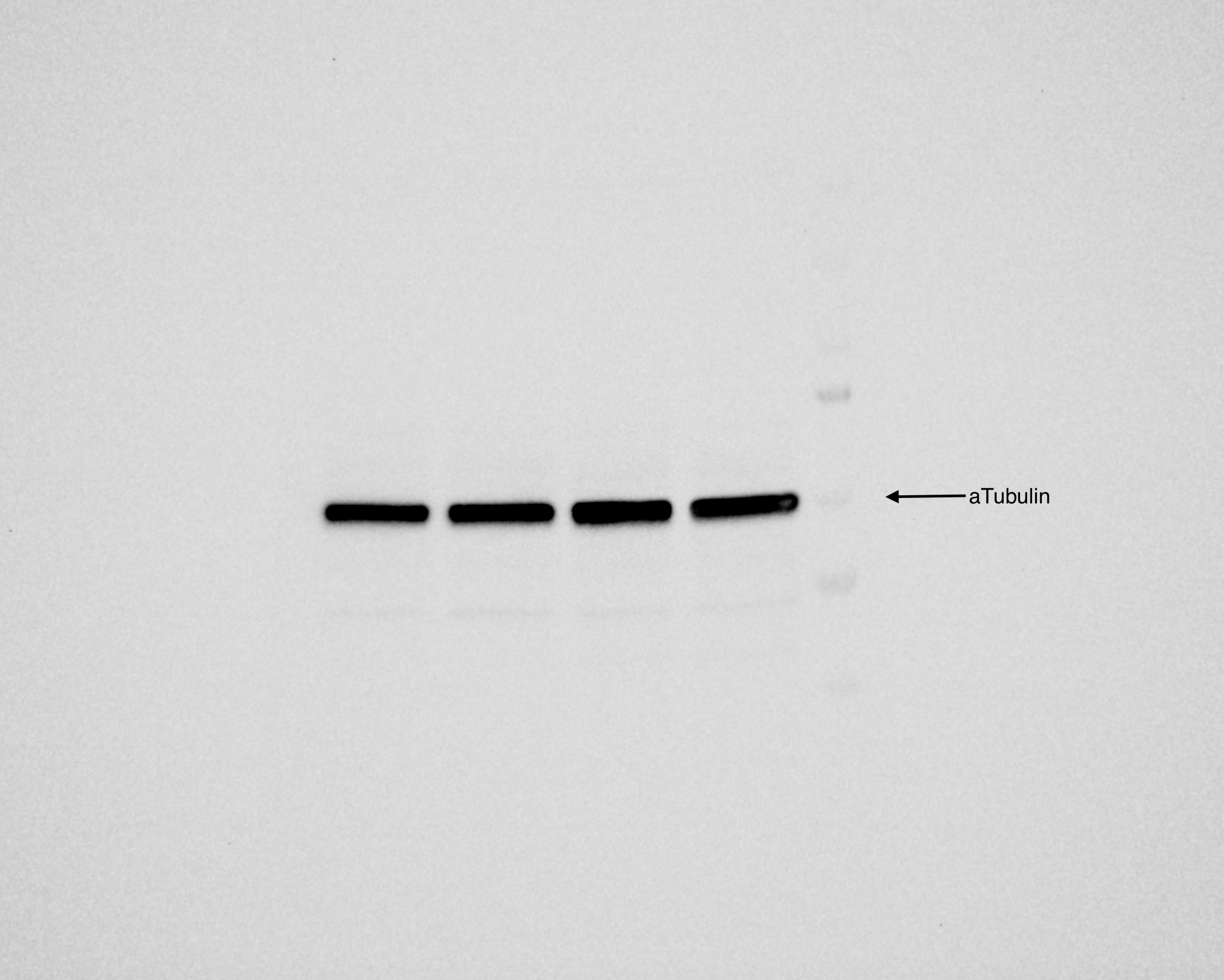

Supplement: Figure 2—source data 3. — Uncropped Western blot images of HK2, LDHA, PHD2, PHD3, and α-tubulin expression in TR-AMs and BMDMs treated with normoxia or hypoxia. [file elife-77457-fig2-data3.zip › Figure 2-source data 3 (Figure 2E)/Figure 2E-aTubulin.tif]

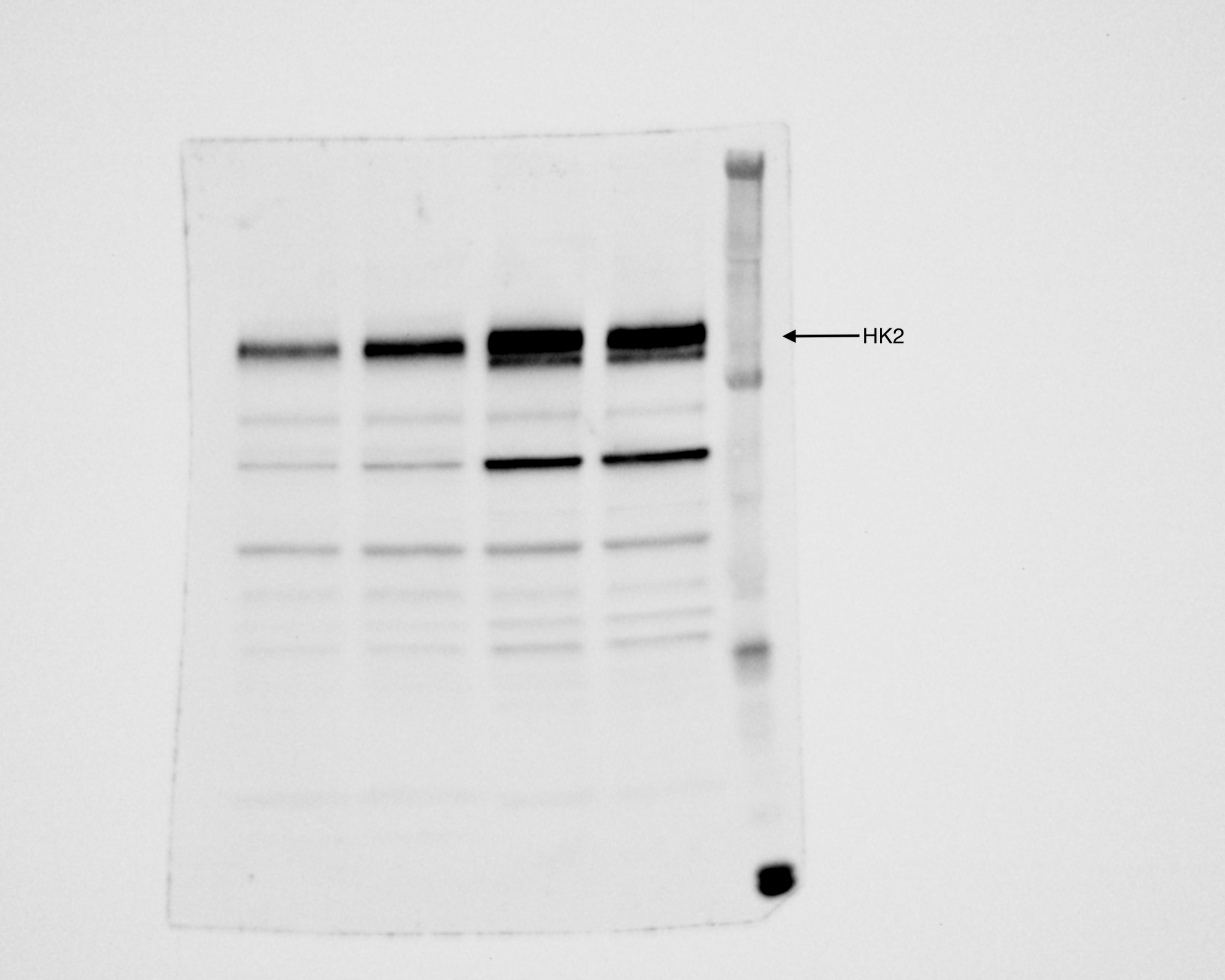

Supplement: Figure 2—source data 3. — Uncropped Western blot images of HK2, LDHA, PHD2, PHD3, and α-tubulin expression in TR-AMs and BMDMs treated with normoxia or hypoxia. [file elife-77457-fig2-data3.zip › Figure 2-source data 3 (Figure 2E)/Figure 2E-HK2.tif]

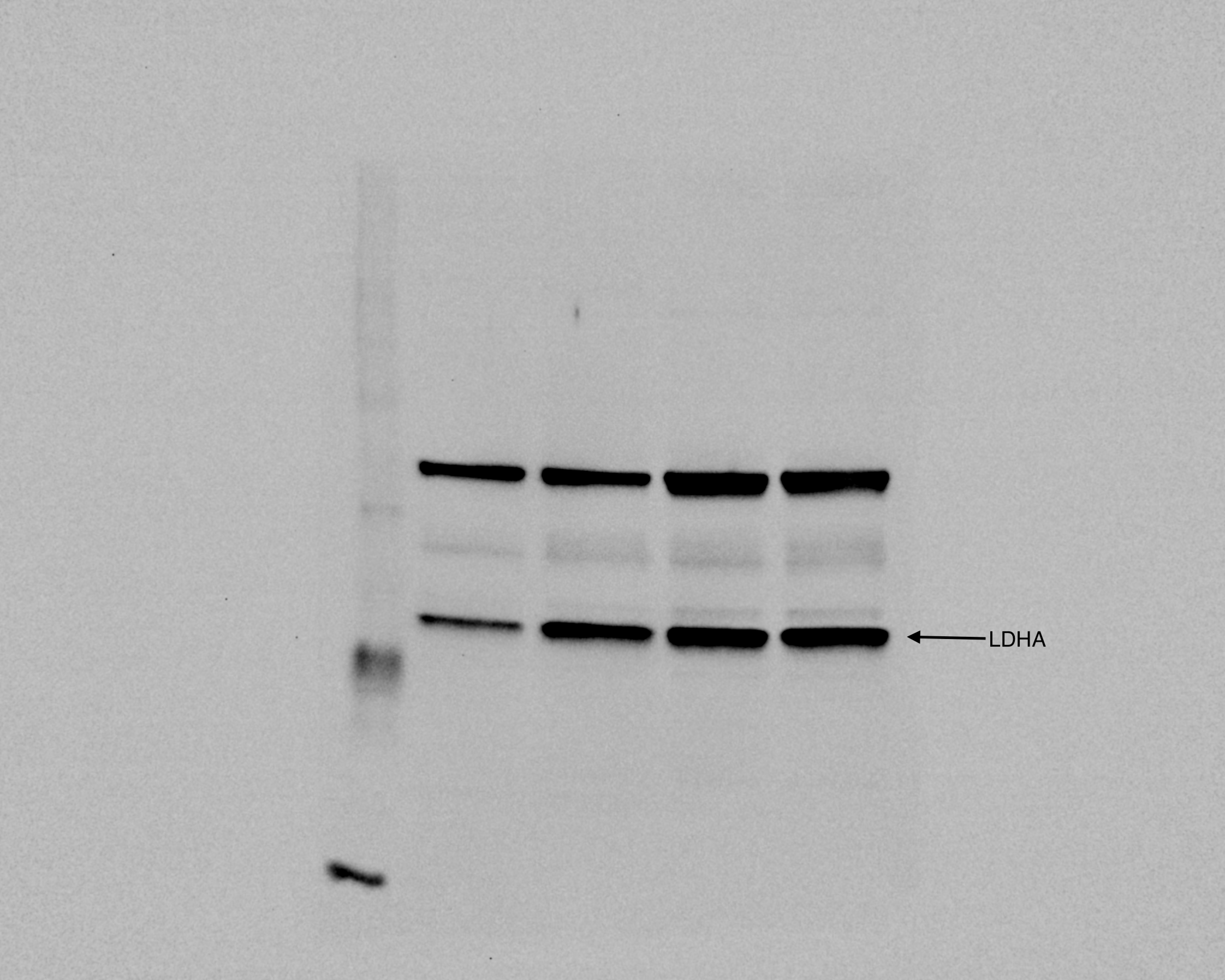

Supplement: Figure 2—source data 3. — Uncropped Western blot images of HK2, LDHA, PHD2, PHD3, and α-tubulin expression in TR-AMs and BMDMs treated with normoxia or hypoxia. [file elife-77457-fig2-data3.zip › Figure 2-source data 3 (Figure 2E)/Figure 2E-LDHA.tif]

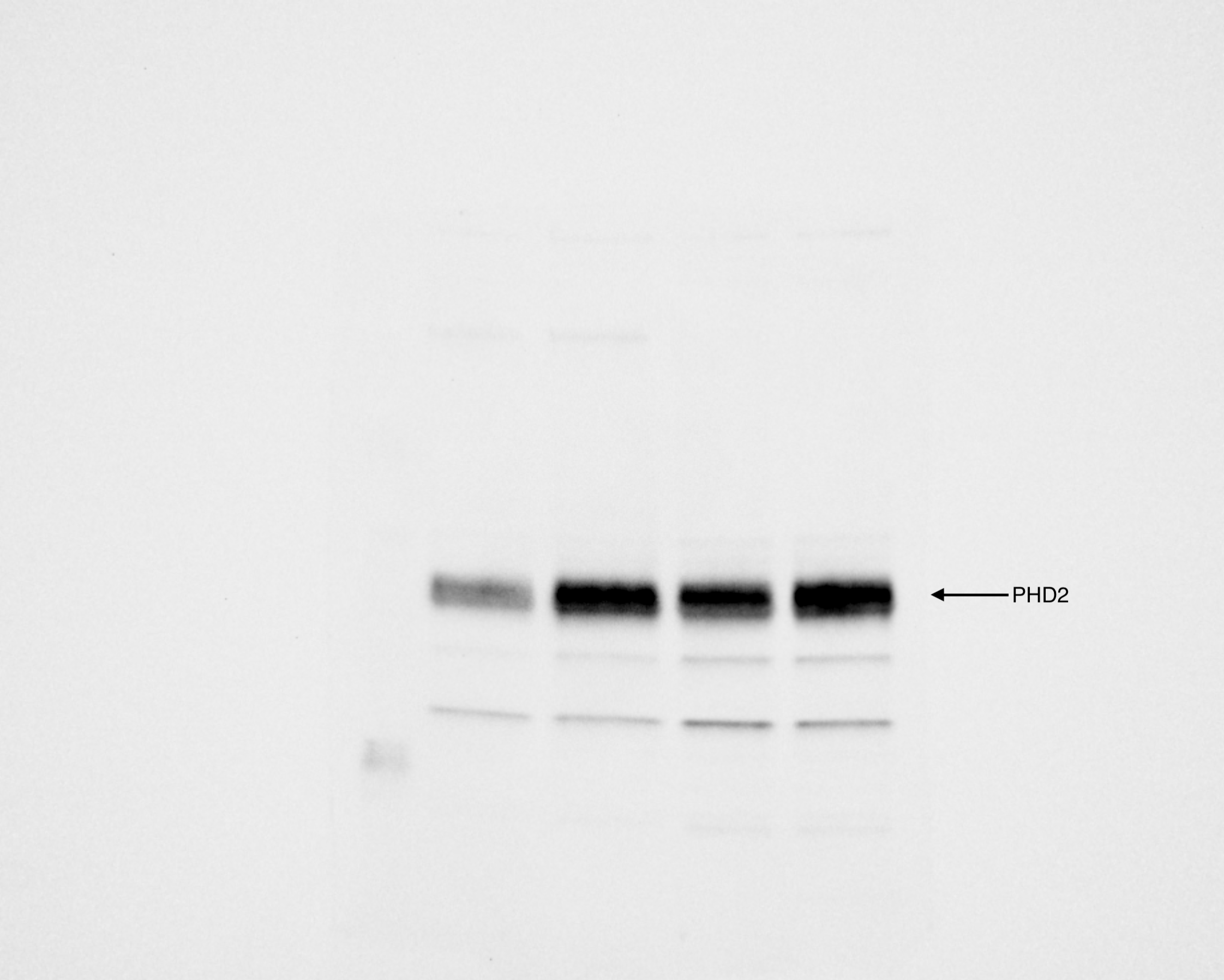

Supplement: Figure 2—source data 3. — Uncropped Western blot images of HK2, LDHA, PHD2, PHD3, and α-tubulin expression in TR-AMs and BMDMs treated with normoxia or hypoxia. [file elife-77457-fig2-data3.zip › Figure 2-source data 3 (Figure 2E)/Figure 2E-PHD2.tif]

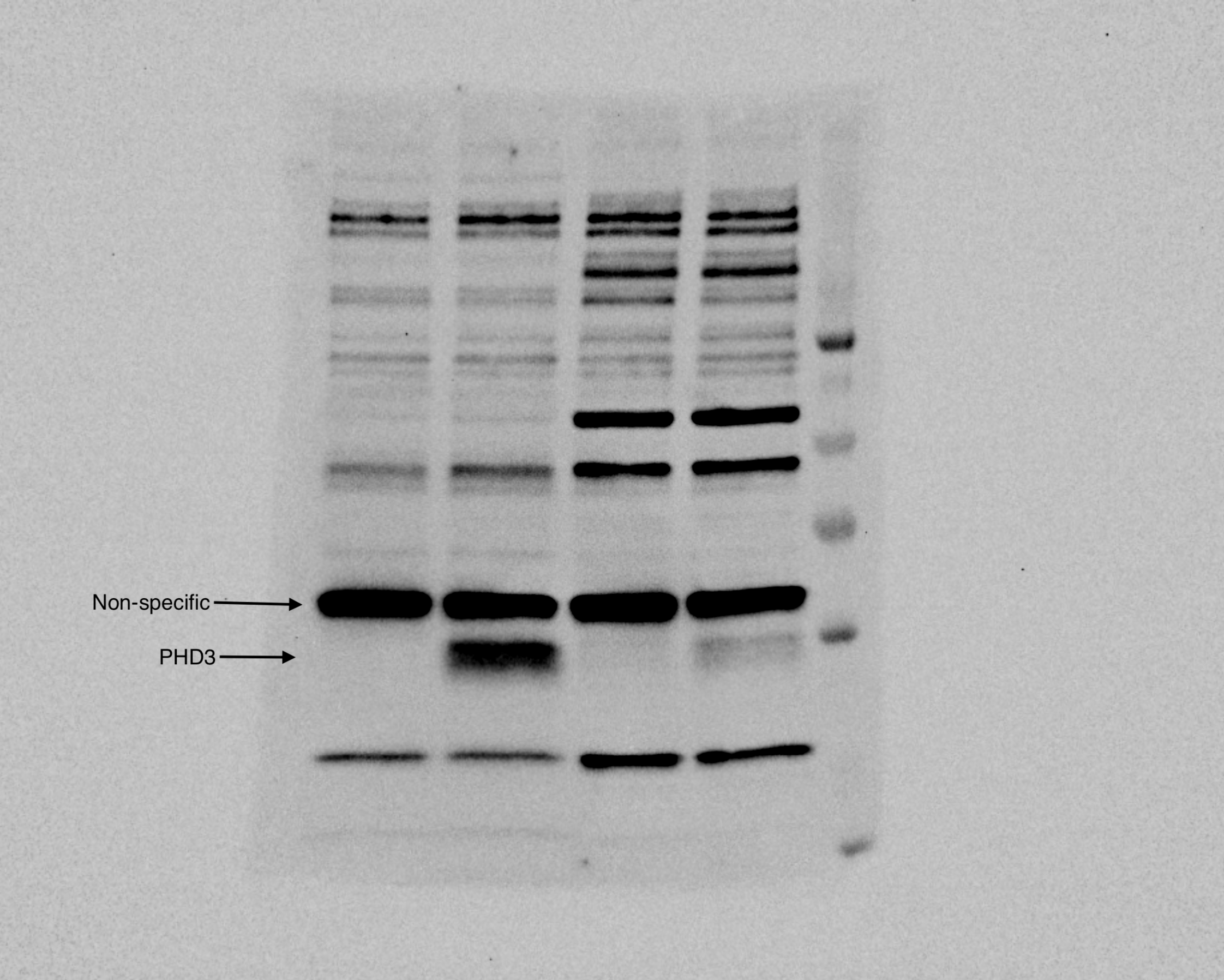

Supplement: Figure 2—source data 3. — Uncropped Western blot images of HK2, LDHA, PHD2, PHD3, and α-tubulin expression in TR-AMs and BMDMs treated with normoxia or hypoxia. [file elife-77457-fig2-data3.zip › Figure 2-source data 3 (Figure 2E)/Figure 2E-PHD3.tif]

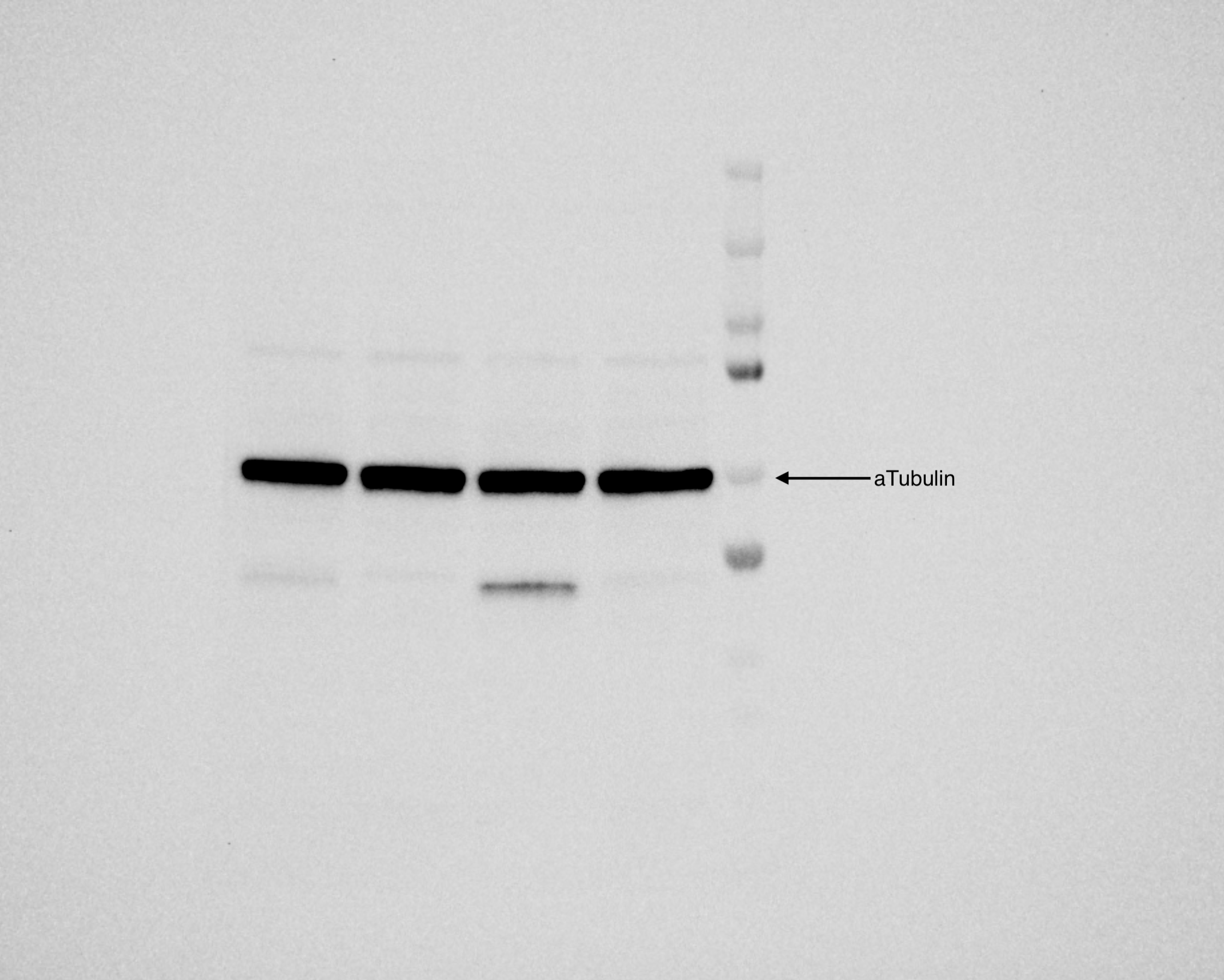

Supplement: Figure 3—source data 1. — Uncropped Western blot images of proIL-1β protein in TR-AMs treated with LPS for 6 or 24 hr under normoxia or hypoxia. [file elife-77457-fig3-data1.zip › Figure 3-source data 1 (Figure 3C)/Figure 3C-aTubulin.tif]

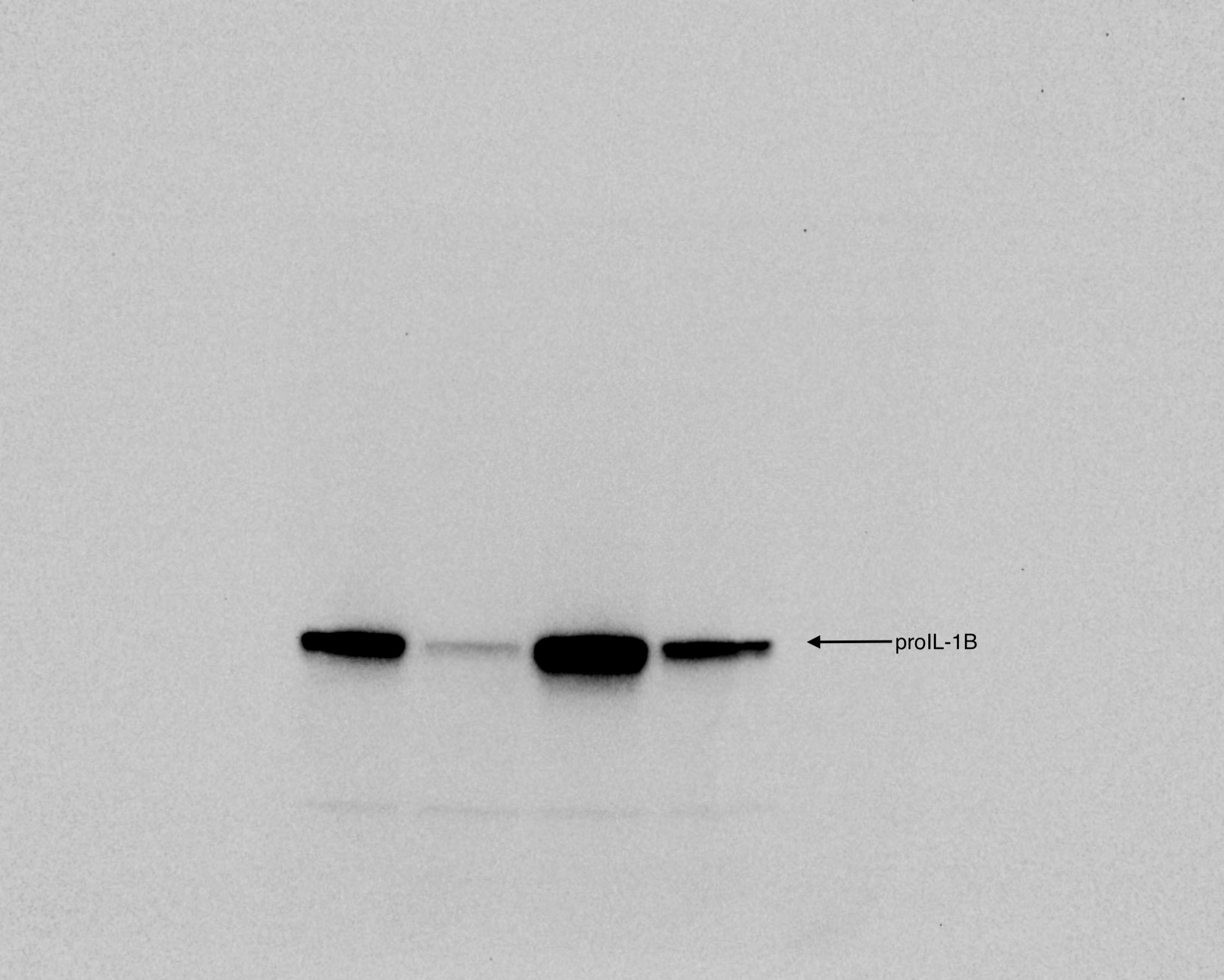

Supplement: Figure 3—source data 1. — Uncropped Western blot images of proIL-1β protein in TR-AMs treated with LPS for 6 or 24 hr under normoxia or hypoxia. [file elife-77457-fig3-data1.zip › Figure 3-source data 1 (Figure 3C)/Figure 3C-proIL1B.tif]
